# A Smartphone App to Assist Smoking Cessation Among Aboriginal Australians: Findings From a Pilot Randomized Controlled Trial

**DOI:** 10.2196/12745

**Published:** 2019-04-02

**Authors:** David Peiris, Lachlan Wright, Madeline News, Kris Rogers, Julie Redfern, Clara Chow, David Thomas

**Affiliations:** 1 The George Institute for Global Health, UNSW Sydney Newtown Australia; 2 Westmead Applied Research Centre, University of Sydney Sydney Australia; 3 Menzies School of Health Research Darwin Australia

**Keywords:** smoking cessation, oceanic ancestry group, mobile apps

## Abstract

**Background:**

Mobile health (mHealth) apps have the potential to increase smoking cessation, but little research has been conducted with Aboriginal communities in Australia.

**Objective:**

We conducted a pilot study to assess the feasibility and acceptability and explore the effectiveness of a novel mHealth app to assist Aboriginal people to quit smoking.

**Methods:**

A pilot randomized controlled trial (RCT) and process evaluation comprising usage analytics data and in-depth interviews was conducted. Current Aboriginal smokers (>16 years old), who were willing to make a quit attempt in the next month, were recruited from Aboriginal Community Controlled Health Services and a government telephone coaching service. The intervention was a multifaceted Android or iOS app comprising a personalized profile and quit plan, text and in-app motivational messages, and a challenge feature allowing users to compete with others. The comparator was usual cessation support services. Outcome data collection and analysis were conducted blinded to treatment allocation. The primary outcome was self-reported continuous smoking abstinence verified by carbon monoxide breath testing at 6 months. Secondary outcomes included point prevalence of abstinence and use of smoking cessation therapies and services.

**Results:**

A total of 49 participants were recruited. Competing service delivery priorities, the lack of resources for research, and lack of support for randomization to a control group were the major recruitment barriers. At baseline, 23/49 (47%) of participants had tried to quit in recent weeks. At 6-month follow-up, only 1 participant (intervention arm) was abstinent. The process evaluation highlighted low to moderate app usage (3-10 new users per month and 4-8 returning users per month), an average of 2.9 sessions per user per month and 6.3 min per session. Key themes from interviews with intervention participants (n=15) included the following: (1) the powerful influence of prevailing social norms around acceptability of smoking; (2) high usage of mobile devices for phone, text, and social media but very low use of other smartphone apps; (3) the role of family and social group support in supporting quit attempts; and (4) low awareness and utilization of smoking cessation support services. Despite the broad acceptability of the app, participants also recommended technical improvements to improve functionality, greater customization of text messages, integration with existing social media platforms, and gamification features.

**Conclusions:**

Smoking cessation apps need to be integrated with commonly used functions of mobile phones and draw on social networks to support their use. Although they have the potential to increase utilization of cessation support services and treatments, more research is needed to identify optimal implementation models. Robust evaluation is critical to determine their impact; however, an RCT design may not be feasible in this setting.

**Trial Registration:**

Australian and New Zealand Clinical Trials Registry ACTRN12616001550493; https://www.anzctr.org.au/Trial/Registration/TrialReview.aspx?id=371792 (Archived by WebCite at http://www.webcitation.org/76TiV7HA6).

## Introduction

### Smoking Rates for Aboriginal and Torres Strait Islander People

Aboriginal and Torres Strait Islander people comprise approximately 3% of the total Australian population [1]. Although there have been recent encouraging declines in smoking overall in Australia, in 2014-15, 39% of Aboriginal and Torres Strait Islander people over the age of 15 years smoked cigarettes daily (36% of females and 42% of males), which is 2.8 times the nonindigenous prevalence [1,2]. Although a smaller proportion of Aboriginal and Torres Strait Islander smokers compared with the general Australian population have ever tried to quit (69% vs 81.4%, respectively) or have ever sustained a quit attempt for more than or equal to 1 month (47% vs 60%), Aboriginal and Torres Strait Islander smokers are just as likely to have attempted quitting in the previous year [3]. There is less social disapproval of smoking among Aboriginal and Torres Strait Islander people when compared with the general population (62% vs 79%), and daily Aboriginal and Torres Strait Islander smokers are less likely to use nicotine replacement therapy (NRT) and other smoking cessation therapies than the general population (37% vs 59%) [3].

### Mobile Health Interventions for Smoking Cessation

Mobile health (mHealth) interventions are emerging as potential strategies to increase smoking cessation. A recent systematic review of 12 studies and 11,885 participants found that smokers who received mHealth support were around 1.7 times more likely to abstain from smoking than smokers who did not receive the programs [4]. Another systematic review examined text messaging interventions for smoking cessation (22 interventions, 10 countries, and 15,593 smokers) [5]. Smokers who received a text messaging intervention were 37% more likely to abstain from smoking relative to controls across several smoking abstinence measures (point prevalence, continuous abstinence, prolonged abstinence, and repeated point prevalence).

There has been only 1 randomized controlled trial (RCT) of an mHealth intervention for smoking cessation in Australia. QuitCoach is a personalized tailored internet-delivered advice program, and onQ (now named QuiTxt) is an interactive automated text messaging program [6]. In a randomized trial comprising 3530 smokers or recent quitters, only 42.5% of those offered 1 of the interventions engaged with those interventions. There were no significant differences between intervention and control groups combined in abstinence rates; however, among those who actually had used an intervention, there was a significant overall increase in abstinence (OR 1.95, 95% CI 1.04 -3.67). In a follow-up study conducted by this group of a smartphone app versus simple text messaging, authors found that the app-based intervention was similar to text messaging in terms of utilization; however, the text messaging system was associated with higher abstinence rates [7].

There have been 2 randomized trials of intensive smoking cessation support for Aboriginal and Torres Strait Islander people in program in remote communities (163 participants) and among pregnant women (263 participants) [8,9]. Although both trials did not demonstrate significant improvement in quit rates, a meta-analysis of data from both trials did show a 2.4-fold increase in quit rates. A systematic review of clinical trials internationally to reduce smoking rates in indigenous populations, conducted in 2013, analyzed 5 studies—3 testing Quitline protocols (structured steps for what happens when a person calls the Quitline including triage, counseling, and follow-up processes) combined with cessation products compared with Quitline alone and 2 using culturally adapted cessation counseling using mobile phones [10]. Outcomes were mixed; however, a New Zealand trial of a text message intervention involving 1705 participants including 355 Maori (the indigenous people of New Zealand) found a greater than 2-fold increase in smoking abstinence at 6 weeks (28% vs 13%), which appeared to be sustained at 6 months [11]. Abstinence rates were equally successful among Maori as non-Maori in the intervention arm [12]. This program is now being administered via New Zealand Quitline.

Contemporary data on mobile phone access for Aboriginal and Torres Strait Islander people were unclear; however, in 2008, 67% of nonremote and 61% of remote households had access to a prepaid mobile phone and 41% and 19%, respectively, had mobile access via a contract [13]. These numbers are almost certainly much higher now. Despite high rates of mobile phone usage, we are not aware of any trials of smoking cessation apps specifically built for indigenous people. Therefore, the aim of this study was to assess the feasibility, acceptability, and preliminary effectiveness of a novel mHealth app to assist Aboriginal people to quit smoking.

## Methods

### Design

**Figure 1:**
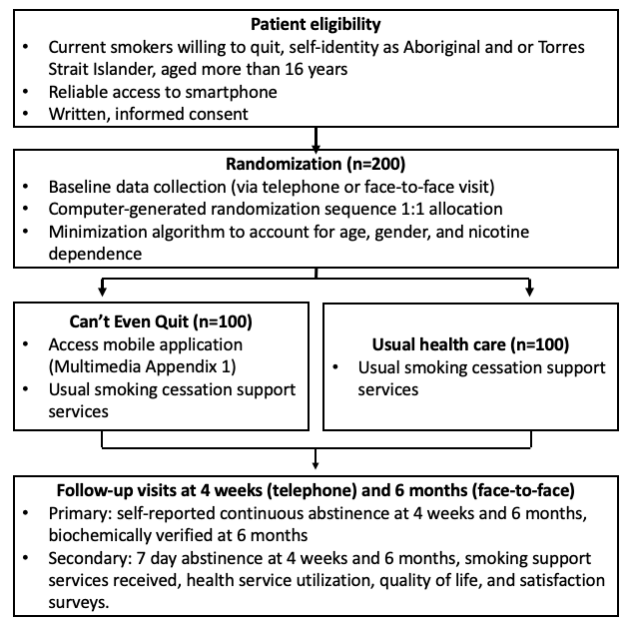
Trial schema.

The pilot study was an RCT (Figure 1). Outcome data collection and analysis were conducted blinded to treatment allocation. The Consolidated Standards of Reporting Trials-EHEALTH statement is available in Multimedia Appendix 1 and the study protocol in Multimedia Appendix 2.

### Participant Eligibility Criteria

Participants were eligible if they could provide informed consent and met all of the following criteria: (1) current smokers aged 16 years or older, (2) self-identification as an Aboriginal and/or Torres Strait Islander person, (3) willing to make an attempt to quit smoking in the next month, and (4) had access to an iPhone or Android smartphone. Only 1 person per household was invited to participate in the study.

#### Recruitment

Recruitment was intended to take place via up to 4 Aboriginal Community Controlled Health Services (ACCHSs), a New South Wales (NSW) government telephone coaching service, and Quitline. Participants were recruited via community events, ad hoc referrals from health professionals, and direct phone calls by research staff based on recommendations made by the ACCHS.

#### Allocation

Randomization was conducted via a central computer-based randomization service. Allocation was 1:1 intervention versus control using a minimization algorithm to balance for sex, age (<30 years vs ≥30 years), and heaviness of smoking index score (low [score≤2] vs moderate or high addiction [score>2]) for nicotine dependence [14]. Outcome analysis and data collection were conducted blinded to treatment allocation.

#### Intervention

**Figure 2:**
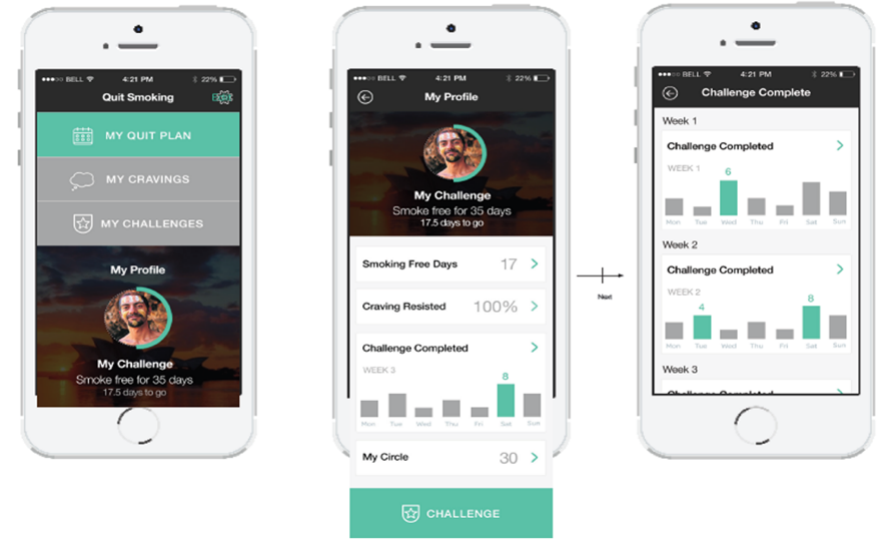
The challenge function screenshots.

**Figure 3:**
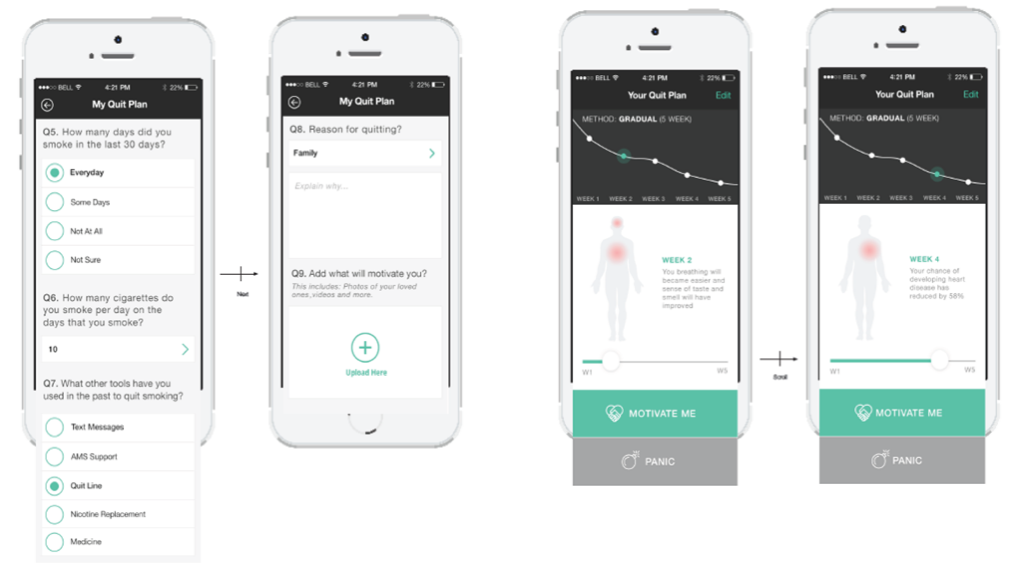
My quit plan screenshots.

**Figure 4:**
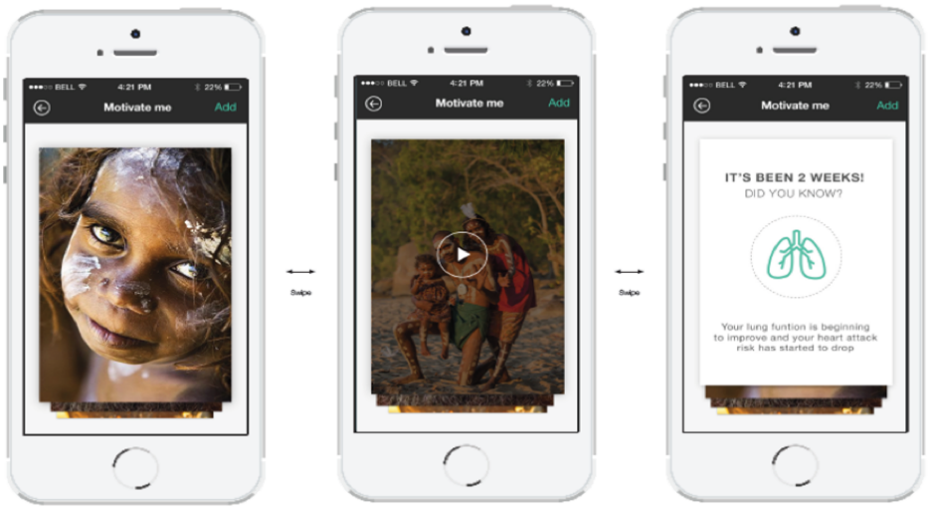
"Motivate me" screenshots.

**Figure 5:**
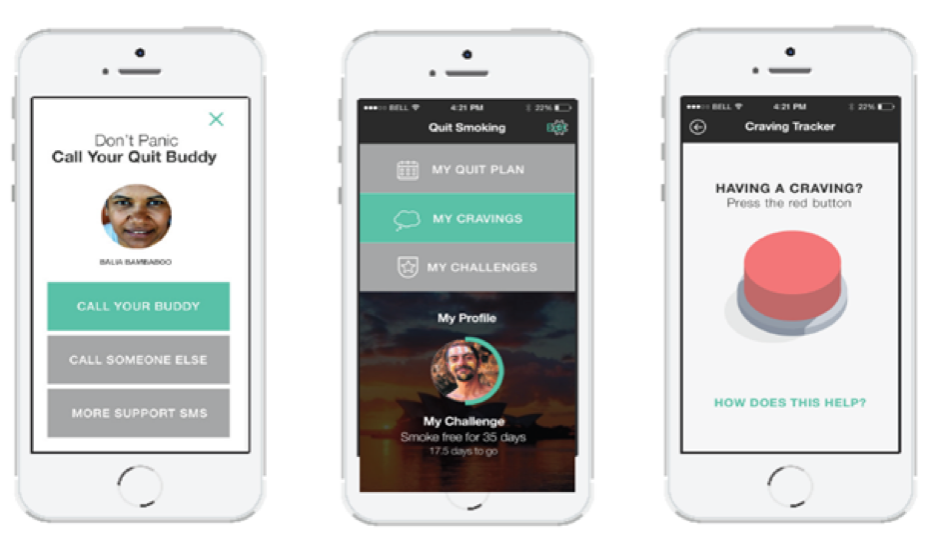
Manage cravings screenshots.

A detailed description of the intervention development, the codesign process, and core features is available in Multimedia Appendix 3. In brief, the intervention was delivered via a multifaceted Android or iOS app comprising a personalized profile and quit plan, text and in-app motivational messages, and a challenge feature allowing users to “compete” with others. Screenshots are shown in Figures 2-5.

All participants were free to use any other smoking cessation service or support and were offered Quitline and local ACCHS contact numbers. Participants allocated to the intervention group were given the opportunity to download and start using the app immediately. There was no cost associated with using the app. A support worker facilitated the registration profile, password set-up and the creation of a tailored quit plan. Participants were then given a tutorial of the app features including how to set up a challenge, motivational support, and tracking progress on their plan. App set up was also done without the use of a support worker as desired.

#### Control

Participants in the control arm and their supporting health services were encouraged to make use of all smoking cessation support services available to them. They were barred from accessing the app if their mobile number matched our database of control arm participants. People not involved in the trial could also download the app if they were invited by an intervention arm participant to use the “challenge” function.

### Data Collection Methods

All study instruments were administered by a questionnaire and an interview. Baseline data collection, consent, and randomization were conducted by telephone or via an in-person visit with a trained project officer. Participants allocated to the intervention were telephoned at 1 week to determine if they needed any support with using the app. There was also a “contact us” number in the app itself to call for help. At 4 weeks, an “evaluation” project officer conducted an outcome assessment by telephone or in-person for all participants in the trial. At 6 months’ post randomization, this officer conducted the final outcome assessment and biochemical verification of smoking status (if needed) as part of a face-to-face visit (Figure 1). In addition to the objective outcome assessment visits, an analytic tool built in the app assessed usage patterns and changes in usage patterns over time.

### Quantitative Outcomes

The primary outcome was self-reported continuous smoking abstinence, objectively verified at 6 months. Self-reported continuous abstinence is defined as the participant reporting they had quit at both 4 weeks and 6 months of follow-up. Self-reported smoking cessation was confirmed with an Airmet Scientific Micro Plus Smokerlyzer (carbon monoxide meter breath test where a reading of less than or equal to 6 represents no recent tobacco smoking) [15]. Secondary outcomes included point prevalence of abstinence and use of smoking cessation therapies and services.

### Statistical Considerations

On the basis of the Text2Quit and Text2Stop trials, we estimated the control arm abstinence rate at 6 months to be 5% [11,16]. Assuming that the abstinence rate is double that of the control arm, we calculated that 1000 participants would be needed to detect a 5% absolute difference (ie, 10.0% vs 5.0%; relative risk 2.00). This assumes a 15% loss to follow-up, 2-sided alpha=.05 and 80% probability of detecting a significant difference with *P* values less than .05 judged as significant. This study was planned as a pilot RCT in which we aimed to recruit 200 participants to assess feasibility, resource considerations, acceptability, and preliminary effectiveness data to inform a future adequately powered trial.

Using the secondary outcome measure of 7-day abstinence rates at 4 weeks and assuming the control arm abstinence rate is 12% and the intervention arm abstinence rate is 2.4 times greater (as observed in the Text2Stop trial), we assumed there would be 80% probability of detecting a significant difference with 200 participants from the pilot study under the same assumptions as above.

Data were analyzed on the basis of intention-to-treat. Characteristics were compared between the groups at 4 weeks and 6-month follow-up using exact Chi-square tests for categorical variables with 2-sided *P* values, where the assumptions of the statistical test could be met. Results were reported in terms of relative risks with exact 95% CI. Analyses were undertaken using SAS 9.4 (SAS Institute, Cary, NC). Given this was a pilot study, no subgroup analyses were prespecified. No imputation techniques were undertaken for missing data.

### Qualitative Evaluation

Semistructured, in-depth interviews with intervention arm participants were conducted to better understand barriers and enablers to smoking cessation in general and to use of the app. Participants were sampled to include interviewees with a range of app engagement levels and a balance of gender and ages. Insights from the Talking About the Smokes (TATS) national survey of attitudes to smoking, quit patterns, and management received were drawn on to guide the interview schedule (Multimedia Appendix 4) [17]. Interviews were conducted both over the phone and in-person and were digitally recorded and professionally transcribed. The first 5 interviews were analyzed by 3 team members, and a preliminary coding framework was derived by consensus. The remaining interviews were then analyzed according to this framework by 2 team members. The thematic framework was iteratively refined as new data and themes became apparent. Interviews continued until no new major themes emerged.

### Governance and Ethics

An executive committee comprising the NSW Ministry of Health (Centre for Population Health and the Centre for Aboriginal Health), Aboriginal Health and Medical Research Council, and The George Institute for Global Health oversaw the project delivery. The Cancer Institute provided ad hoc advice and reviewed progress throughout the project. An expert user group was convened early in the project and regularly consulted during the intervention development phase (Multimedia Appendix 2). Ethical approval was granted by the Aboriginal Health and Medical Research Council Human Research Ethics Committee. Formal approvals from each of the participating sites were also granted. Due to an administrative oversight, the trial was inadvertently registered retrospectively after the first participant was enrolled in the trial.

## Results

### Recruitment

We were only able to collaborate with 1 ACCHS, 1 regional community event, and the NSW government coaching service to assist in recruitment. Furthermore, 3 ACCHSs decided against participation. The major barriers to participation included the following: (1) competing service delivery priorities, (2) lack of resources for research, and (3) preference that the intervention be made available to all interested clients of the service rather than random allocation to intervention and control arms. Due to a change in the contracted service provider for Quitline during the trial, we were also unable to negotiate an agreement with the new provider within the project timelines.

**Figure 6:**
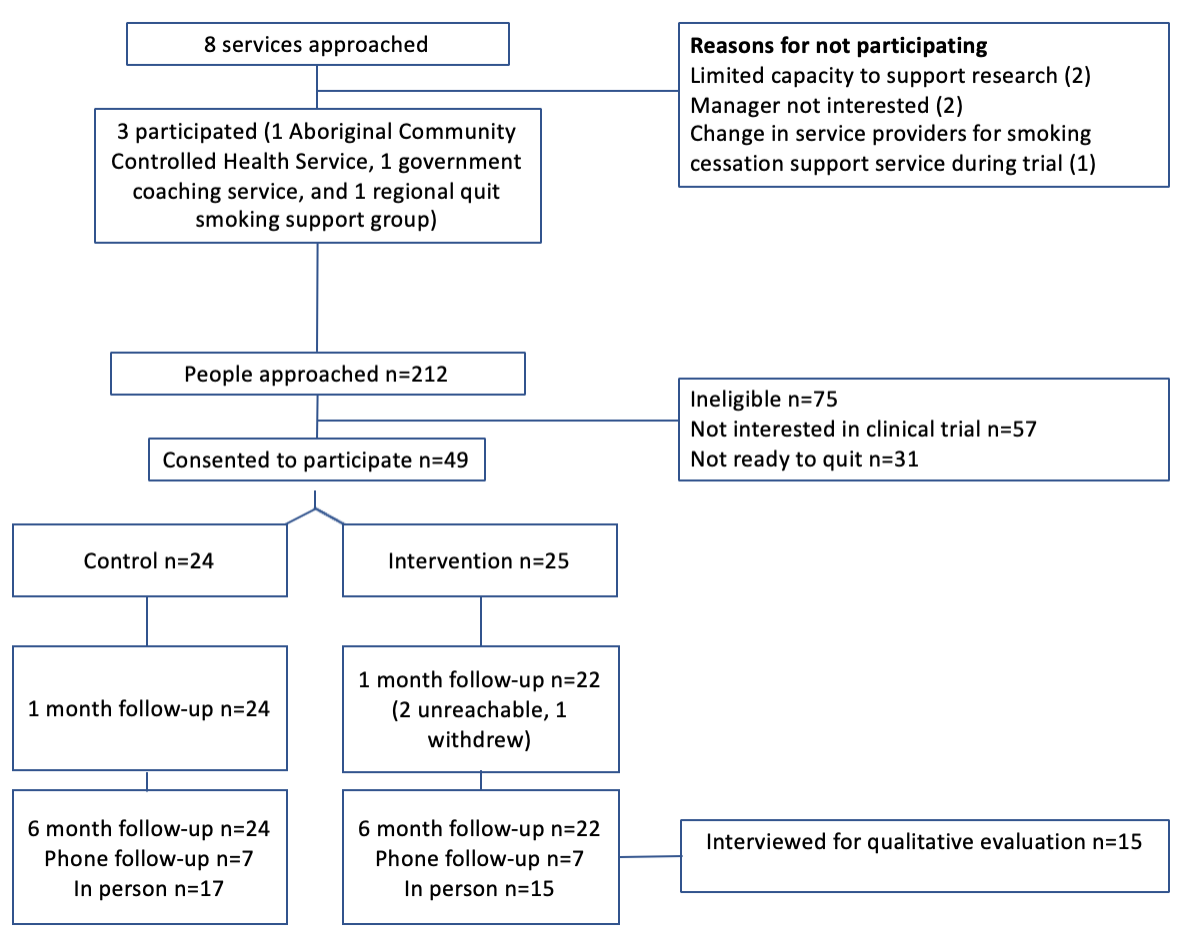
Study flow.

Due to these recruitment challenges, only 49 of the target 200 participants were recruited between March 2016 and January 2017, with the majority recruited from 1 ACCHS (Figure 6). There were no changes made to the trial outcomes after the trial commenced.

Table 1 shows the baseline characteristics of the trial participants by randomization arm. Broadly, both groups were similar. The majority of participants in both arms had made at least one quit attempt in the previous 12 months and almost half had tried to quit in recent weeks (23/49, 47%). The majority of participants (39/49, 80%) smoked their first cigarette of the day within 30 min of waking. Most participants (44/49, 90%) commenced smoking before the age of 20 years. Most people lived in households where at least one other person smoked (33/49, 67%) A minority had made use of support services, medication, or NRT. Most participants had seen a doctor in the previous 12 months. In terms of use of mobile technology, most participants used their mobile phone for more than 30 min a day for voice calls (29/49, 60%), text messages (31/49, 64%), smartphone apps (35/49, 72%), internet (37/49, 75%), and social media (41/49, 83%).

**Table 1:** Participant baseline characteristics.

Characteristic		Intervention (n=25)	Control (n=24)	Total (n=49)
Female, n (%)		19 (76)	19 (79)	38 (78)
Male, n (%)		6 (24)	5 (21)	11 (22)
Aboriginal, n (%)		25 (100)	24 (100)	49 (100)
Age (years), mean (SD)		42 (14)	42 (14)	42 (14)
Smoke every day (or nearly every day), n (%)		25 (100)	23 (96)	48 (98)
**Number of smokes per day, n (%)**				
	10 or less	6 (24)	8 (33)	14 (29)
	11-20	15 (60)	9 (38)	24 (49)
	21-30	3 (12)	6 (25)	9 (18)
	31 or more	0 (0)	1 (4)	1 (2)
**Level of addiction^a^, n (%)**		0 (0)	1 (4)	1 (2)
	Low	8 (32)	8 (33)	16 (33)
	Moderate	15 (60)	15 (63)	27 (55)
	High	1 (4)	1 (4)	5 (10)
**Highest level of formal education or employment, n (%)**				
	Some high school (no certificate)	8 (32)	6 (25)	14 (29)
	Completed high school	2 (8)	5 (21)	7 (15)
	Technical education certificate	11 (44)	8 (33)	19 (40)
	Some university (no degree)	1 (4)	4 (17)	5 (10)
	Completed university degree	1 (4)	0 (0)	1 (2)
	Postgraduate degree	1 (4)	1 (4)	2 (4)
	Paid employment	13 (52)	10 (42)	23 (48)
**Current mobile phone used, n (%)**				
	iPhone	8 (32)	9 (38)	17 (35)
	Android	16 (64)	15 (63)	31 (65)
**Mobile phone subscription plan, n (%)**				
	Prepaid	14 (56)	15 (63)	29 (60)
	Regular plan	10 (40)	9 (38)	19 (40)
**Quit patterns, n (%)**				
	Number of people who had a previous quit attempt in the last 12 months	21 (84)	21 (88)	42 (88)
	Use of any type of nicotine replacement therapy or other stop-smoking medications in the last year	5 (20)	10 (42)	15 (31)
	Sought any information or quit services in the last 12 months	1 (4)	5 (21)	6 (13)
	Previously used Quitline	0 (0)	0 (0)	0 (0)
	Local quit program	1 (4)	0 (0)	1 (2)
**Seen a health worker, doctor, nurse, or other health professional in the last year, n (%)**				
	Doctor	18 (75)	21 (88)	39 (82)
	Nurse	5 (20)	6 (25)	11 (23)
	Aboriginal health worker	3 (12)	3 (13)	6 (13)
	Tobacco worker	1 (4)	1 (4)	2 (4)

^a^Using the heaviness of smoking index where a score of 0-2 indicates low addiction, 3-4 moderate addiction, and 5-6 high addiction.

### Trial Outcomes

Table 2 shows the primary outcomes of the trial for the 46 people who completed the study. Only 2 participants (intervention arm) reported abstinence at either the 4-week or 6-month interview, and nobody had continuous abstinence at both time points. There were generally higher numbers of intervention arm participants reporting the use of supportive cessation services, but these were not statistically significant. There were no harms or unintended consequences observed in this study.

### App Usage Analytics

**Figure 7:**
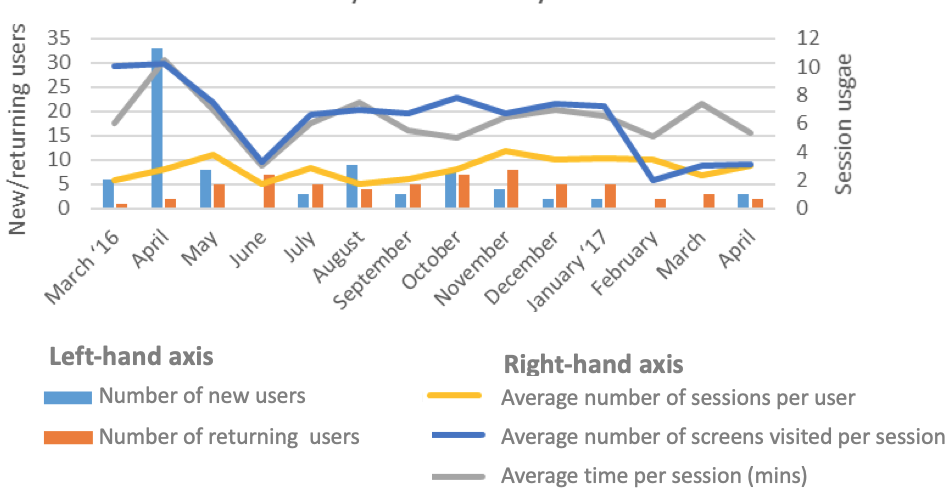
App usage by month (March 2016-April 2017).

The usage data for the app from March 2016 to April 2017 are shown in Figure 7. These data include both intervention arm participants and nontrial participants who were invited to use the app as part of the challenge function (81 new users in total for the trial period). From June to December 2016, there was a relatively consistent rate of usage in terms of new users (3-10 users per month) and returning users (4-8 users per month). Session frequency average was 2.9 times per user per month, average session time was 6.3 min, and the average number of unique screens viewed per session was 7.6. These statistics indicate a low to moderate level of usage through the trial period.

**Table 2:** Trial outcomes.

Outcome^a^		Intervention (n=22)	Control (n=24)
**Self-reported smoking abstinence, n (%)**			
	At 4-week visit	1 (4.5)	0 (0)
	At 6-month visit (verified with breath test)	1 (4.5)	0 (0)
	At both 4-week and 6-month visit	0 (0)	0 (0)
	Self-reported quit attempt since starting the trial	5 (23)	6 (25)
**Use of other smoking cessation services during the trial, n (%)**			
	Any nicotine replacement therapy use during study	5 (23)	5 (21)
	Any smoking-related medication use	4 (18)	2 (8)
	Quitline or any other quit program	2 (9)	0 (0)
	Any combination of the above cessation aids	9 (41)	7 (29)

^a^The total number of participants who achieved 1 of the main or secondary outcomes was small, so we did not perform statistical testing or calculate a relative risk ratio as we could not meet the assumptions required.

**Figure 8:**
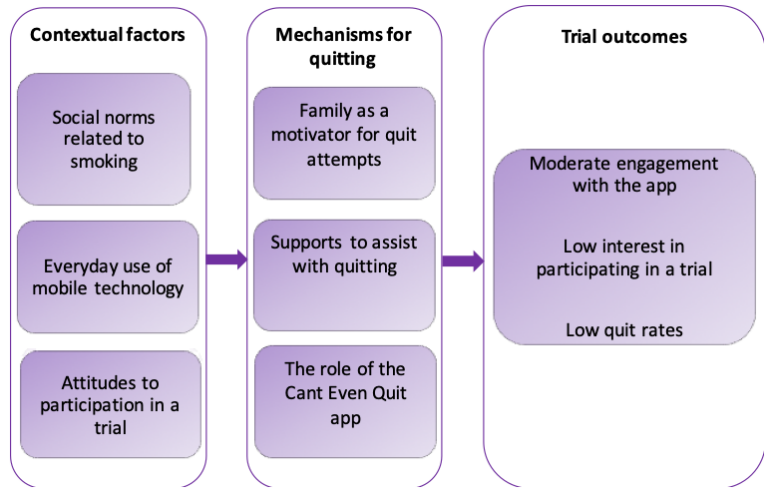
Qualitative themes and their influence on the trial outcomes.

### Interview Findings

A total of 15 interviews with intervention arm participants were conducted, ranging from 20 to 45 min duration. Furthermore, 6 major themes arose from the analyses of these interviews. These are illustrated in Figure 8 using a context, mechanism, and outcome configuration to demonstrate the potential influence of these themes on smoking cessation and to assist with explaining the outcomes observed in the trial. More descriptive detail on the context- and mechanism-related themes is provided below.

#### Contextual Factors Influencing the Trial and Outcomes

##### Theme 1: Social Norms Related to Smoking

All trial participants had commenced smoking before the age of 18 years. Interviewees described living in households where smoking was the norm among adults and how these norms strongly influenced children:

You’d see the plumes of smoke in the kitchen...There were ashtrays all over the place...we used to play with the butts and pretend to smoke them ourselves

female, 61 years

Another participant described the strong influence of girls’ peer groups in driving the commencement of smoking describing how she:

Would go home and my friend would give me one out of her mum’s packet and it went on from there.

female, 44 years

Another participant described being paid in cigarettes for babysitting for his neighbor. The consequence of such entrenched normalizing behaviors was that smoking was viewed as a natural aspect of progression to adulthood with 1 participant commenting:

I got my first pay check at 14 and I bought a packet of cigarettes.

female, 39 years

##### Theme 2: Everyday Use of Mobile Technology

Most participants cited phone usage of some kind. Facebook was one of the most popular features with 1 participant describing it:

Like the fridge, you’re always opening it.

female, 44 years

Only 2 participants had used a health-related app (unrelated to the study app). For these participants, there was an initial curiosity to explore the features and its potential to assist with one’s health, but this tended to be short-lived:

I downloaded it, but then, yeah, it was a lot of rubbish so I took it off.

female, 48 years

Most older interviewees did not appear to have any major issues with knowledge on how to access phone features. However, most people rarely made use of smartphone apps apart from those used for playing games. Moreover, 1 participant commented:

...I play them (game apps) on the way to work...maybe three hours a day.

male, 24 years

Furthermore, 1 interviewee suggested that game apps could provide a stronger motivation for engaging in health apps.

##### Theme 3: Attitudes to Participation in a Trial

A study design involving a control arm that did not receive the app was not viewed favorably, given this was a cohort of current smokers who had expressed the intention to quit smoking within the next month:

I seen it [the trial] promoted on Facebook...and a couple of posters in the waiting area, and I was actually encouraging a lot of patients to come down for it, but I didn’t know it... [depended on]... whether you got randomized. I was glad I was in the trial [intervention arm], because I would have been frustrated if I wasn’t.

female, 44 years

This sentiment was also expressed by several ACCHS managers. Although the decision to participate was influenced by a range of factors, some managers were uncomfortable with both the resource burden of participating in a trial and the lack of ability to provide the intervention as a part of routine service delivery. Many expressed interest in participating in a posttrial phase once the RCT was completed.

#### Mechanisms for Quitting

##### Theme 4: Family as a Major Motivator to Quit Attempts

Although social norms were described as strong drivers in initiating and maintaining smoking, similarly many participants cited family as being the major motivator for quitting:

So what I want...is when I cuddle my girls they’re not saying, “Oh Nanny, you smell like cigarettes”...that breaks my heart. And it makes me more determined...I want them to remember me as their nanny who loved them very much...

female, 61 years

Although many participants had used smoking cessation aids, key events such as the death of a relative appeared to be critical moments in supporting quit attempts:

Losing my mum two years ago has really, really made me want to quit.

female, 44 years

The benefits of being smoke-free were also frequently framed in the context of improving family well-being:

I could use that (money from cigarettes) for something better, for another cruise where I can take the girls...if I save $25 every time I buy a smoke, I might have enough to go on a world cruise. It could happen.

female, 44 years

##### Theme 5: Supports to Assist With Quitting

Around one-half of the participants had previously attempted a range of smoking cessation methods to help them quit, in particular, NRT and mFedication. In addition to these support strategies, several participants mentioned the value of a group atmosphere to support smoking cessation:

...a support group, like AA...sharing ideas, so you encourage each other a bit more.

female, 44 years

Another participant suggested:

You’d have each other to lean on and to express what you’re feeling.

female, 39 years

Some commented on how an app could extend this group support further:

...then you could send this person a message, “Mate, I’m having a downer.”...And if they’re on the other end they can give you some positive feedback.

female, 48 years

Although all participants had heard of Quitline and some had been referred to the program, no one reported actually using the telephone service. Some participants expressed problems with the referral process and this led to a loss of confidence in the service:

I don’t think they rang me...I don’t know if they’re just tokenistic.

female, 44 years

The same participant suggested that the fact that the referral was coming from a third party may have signaled a lack of motivation:

Maybe if you don’t do it yourself (self-refer), they don’t take it as seriously.

##### Theme 6: The Role of the Can’t Even Quit App

###### Technical Difficulties

Although most participants found the app intuitive and easy to use, some participants would have benefited from more extensive training. For example, 1 participant was not aware of the ability to alter the quit date in the app settings. Given the app was designed to provide tailored content according to the user’s quit status, this caused problems:

I stuffed up my start date...to start giving up smoking. And then, it flashed something up, and I’m thinking, “I haven’t quit yet, what are you sending me these for?” ...and that messed everything up. And probably just another excuse not to give up.

female, 44 years

Several participants indicated interest in the challenge function both for oneself and to challenge others; however, there were a range of technical issues that limited its usage such as halting a current challenge and initiating a new one and inviting new users to engage in a challenge. Other technical barriers related to difficulties downloading the app over a cellular network and an automatic log out requiring the user to sign in again after 3 days of inactivity. For a minority of people, these issues substantially limited use of the app.

###### An Intrusive Versus Supportive Service

A positive feature of the app was the support provided by trial staff in using the intervention. Several participants appreciated the phone call follow-ups:

I’d say keep the ringing up. As much as us smokers dodge people that’s because it is a hard subject and we do need that push and encouragement.

female, 44 years

Although this level of “intrusiveness” from the trial staff was generally viewed favorably, some interviewees warned of the risk of “overkill.” Moreover, 1 participant highlighted the need to strike a balance:

I was part of that NSW Health service...Get Healthy. Oh and you’d dodge them like a bullet! They were painful...So once I see your number, I go, “Oh, THEM again!” I think you’ve got to find the right balance.

female, 44 years

This was particularly an issue with the motivational text messages. For some participants, the high message frequency (Multimedia Appendix 2) was viewed positively, and when the frequency reduced, they started to disengage:

I was getting texts all the time, reading all of them, trying to take in all the information...They were good...motivating, especially when you’re having a hard day and you get texts all the time, and it’s like, “Yeah, I can do it.” ...In the beginning I was getting about five or six [text messages], and then it just petered off...

female, 46 years

Others found the text message content to be initially useful, but that they would start to lose their appeal over time:

I started ignoring them. They went into a dark place.

female, 46 years

Some suggested the messages needed to be more engaging over time:

It was exciting at first, but...I want fun stuff...

female, 48 years

Others found the messages came too frequently and this detracted from the personalized nature of these messages:

One time I got five in the space of three and a half minutes, and that’s when it felt to me it was just automated.

female, 44 years

###### Other Suggestions for Improvement

Many commented on the value of incorporating games into the app:

There were no games that you could play...like my jigsaw puzzle on my iPad, there was nothing ...[where] you could go “Oh yeah, I might have to do a little quiz.

female, 48 years

Several participants were regular Facebook users and most of these users felt the app could have been more social, interactive, and inclusive of user-generated content:

...Bring more people to it...maybe put funny little videos or...more stories where you can link in with people...Have it open.

female, 48 years

Addition of an enhanced social element and linkage to support services such as Quitline was seen as essential to harnessing greater interest in the app:

...if they (Quitline) can see that you are having a hard time, then maybe you can get a support call from them...Because...there wasn’t enough people for me to join to become friends with. So you’re sort of isolated on there.

female, 48 years

## Discussion

### Principal Findings

This study outlines the development, implementation, and pilot evaluation of an mHealth strategy to support Aboriginal people with smoking cessation. The evaluation shed valuable information on the challenges of implementing an RCT in this setting and clearly raises serious questions about the feasibility of conducting a larger-scale trial. Despite these challenges, it also shed light on the future potential for mobile apps in this area. We discuss the findings and implications across 3 broad areas: (1) the population, (2) the setting and social context, and (3) this particular app and lessons for other apps in this setting.

The population which participated may represent a “hard-to-reach” group in terms of cessation support services and consequently may differ from the types of populations that have participated in previous mHealth quit smoking trials. A large number of female participants were enrolled in the trial, and analyses from systematic review data suggest that mHealth smoking cessation interventions may be less effective for women than men [5]. Consistent with other studies of quitting among Aboriginal and Torres Strait Islander people, we found that few participants successfully sustained abstinence, used NRT, smoking cessation medications, or cessation services [3,18,19]. These factors suggest that a “one app fits all” approach is unlikely to be successful and a better understanding of the needs and opportunities for groups that have difficulty sustaining quit attempts is needed. A recent international critical review of the literature relating to Indigenous people’s use of health technologies found that such technologies required meaningful user involvement, community-based processes for development, and creative adaptation to local needs [20].

The importance of the setting and social context in which people smoke was a prominent finding from the interviews. Several interviewees commented that social norms around smoking are key drivers of smoking rates in the community. It has been suggested that changes in these norms have occurred more slowly in Aboriginal and Torres Strait Islander communities, and this may be a key factor in the differential decline in smoking rates compared with the general population. Our interviews highlighted many examples of social influences where participants were exposed to smoking behavior early in life and the role this may have played in initiation and maintenance of smoking [21]. A social network analysis study of Aboriginal people in the Australian Capital Territory found separate clustering of smokers from nonsmokers in the social network and that the proportion of adults in a person’s household who smoked was associated with being a smoker [22].

Despite historically high levels of social acceptability for smoking in Aboriginal communities, this is clearly changing. Some have suggested that social contagion of behaviors such as quitting rely on changing norms [23]. The TATS study found that attitudes and beliefs positively associated with wanting to quit included regretting ever starting to smoke, perceiving that local Aboriginal and Torres Strait Islander community leaders disapprove of smoking, believing nonsmokers set a good example for children, worrying about future smoking-related health effects, and believing quitting to be beneficial [3]. We also found in the interviews that although families can influence initiating smoking, they can also be a highly motivating influence to quit smoking. Several participants interviewed commented on how key family members viewed smoking as undesirable, and this was a strong driver for making quit attempts. Others said they would like group support and a group atmosphere to support them in their smoking cessation journeys.

The above 2 findings related to population and social context have implications for future app development considerations. Although most participants cited phone usage of some kind (mainly texting, phone calls, and Facebook), smartphone apps were not widely used, and only 2 participants had used a health-related app previously. The usage analytics showed modest but sustained usage over the life of the study. Many participants, particularly female, recommended that the Can’t Even Quit app needed to be socially “opened up” like Facebook so that more stories could be shared. Aboriginal and Torres Strait Islander people may use Facebook at higher rates than the general Australian population, and a recent qualitative study highlighted potential opportunities for leveraging Aboriginal and Torres Strait Islander people’s use of social media departing from traditional one-way information models of health promotion to assets-based, self-empowerment models [24]. If smoking cessation apps could access a person’s social network, they may be able to facilitate support from network members and spread an intervention through these networks [25]. Currently, most health apps that offer Facebook integration are related to exercise tracking and calorie counting. A 2014 review found only 9 Facebook-integrated smoking cessation apps and these were of variable quality [25]. Pediatric researchers have started to explore Facebook communities for children with chronic diseases and proposed 3 considerations for how this platform could be better leveraged for health issues [26]. These include the following: (1) “back end” content management systems mediated by trusted health professionals that can be integrated with Facebook, (2) tools that can extract data from Facebook feeds and providing structured threads and forums to promote topic-specific information exchange between users, shifting dialogue from “one to many” to “many to many”; and (3) using the platform for consultation and codesign processes when developing health integrated apps.

Clearly, social media apps are only 1 strategy for strengthening social networks related to health issues. A review of the Australian government’s Tackling Indigenous Smoking and Health Lifestyle program highlighted the need for multilevel approaches [27]. In addition to social media and community-based campaigns, health service strategies that are locally tailored, maximize community participation, and strengthen workforce capacity are needed. In this study, the broad appreciation for regular support from research staff suggests that the model of “prescribing” the app as part of a coordinated smoking support service package may be attractive. Given the self-reported rates of seeing health professionals were high (88%) in this study and consistent with previous literature, strengthening health service–mediated interventions should be pursued further [18]. Although we had intended to better understand the role of the app as part of the Quitline service, our inability to recruit participants via this means precluded being able to address this. Questions remain regarding the optimal level of support that is needed with such services. A key finding from the interviews was the need to balance intrusiveness versus supportiveness, particularly for text messages. The evidence on the optimal dose of message frequency is unclear, and further research to test different intensities are needed to help answer this question.

### Limitations

Although we were not expecting to show statistically significant changes in the primary outcome of this pilot, we did intend to generate sufficient preliminary data to inform a large-scale, well-powered trial. Unfortunately, the study fell short of the target numbers for recruitment. On the basis of previous trials, we calculated that over 1000 participants would be needed for an adequately powered study. The overall abstinence rates at 4 weeks and 6 months in this study were lower than the hypothesized 5% quit rate estimated from the literature. This indicates that quit rates may be lower than assumed, and therefore, an even greater sample size may be needed for a fully powered RCT. Our difficulties in achieving recruitment targets were multifactorial, and barriers occurred at both institutional and individual participant levels. More effort will also be needed to attract higher numbers of men into future research of this nature, as many of the findings presented here may reflect gender-specific views. Although we had success implementing the trial at 1 ACCHS, this was not true for several other sites that we approached. For ACCHSs, competing interests, the lack of dedicated resources to support a research study, and a perceived challenge in “selling” a trial where only half of the participants would be given the intervention were the major barriers [28]. Indeed, many ACCHS managers indicated a strong interest in accessing a program if it were made available in a nontrial setting. Alternative evaluation and alternative designs that maintain methodological rigor, enhance institutional and participant acceptability, and place a limited resource burden on health services are needed. Potential options include testing multiple interventions using a factorial RCT design as was done in the Victorian RCT [6], embedding periodic outcome assessments into the app itself and constructing matched control groups from routinely collected datasets.

### Conclusions

This pilot of a smoking cessation app for Aboriginal people was difficult to implement, and few conclusions can be made regarding its effectiveness. Despite this, many insights were gained from the evaluation that bear consideration for future research in this space. In relation to health apps, the findings underscore the importance of a socio-technical approach to their development, comprehensively understanding and embracing the complex interaction between people, their social contexts, and rapidly changing technologies. Although maximal benefit would come from working with people that experience the greatest difficulty with sustaining a quit attempt, it is important to recognize that this is a heterogeneous group that may have particular needs that are not being addressed through existing interventions. Finally, to enhance adoption of such apps, we suggest that a 2-pronged approach is needed that focusses on both service delivery models that promote app “prescription” as part of an overall comprehensive smoking cessation support service and health promotion strategies that support community empowerment.

## Appendix

Multimedia Appendix 1 CONSORT‐EHEALTH checklist (V 1.6.1).

Multimedia Appendix 2 Study protocol.

Multimedia Appendix 3 Development of the intervention.

Multimedia Appendix 4 Participant interview guide.

## Abbreviations

ACCHS: Aboriginal Community Controlled Health Service
mHealth: mobile health
NRT: nicotine replacement therapy
NSW: New South Wales
RCT: randomized controlled trial
TATS: Talking About the Smokes
