# Supplementary material for: A Smartphone App to Assist Smoking Cessation Among Aboriginal Australians: Findings From a Pilot Randomized Controlled Trial
Source: JMIR Mhealth Uhealth. 2019 Apr 2;7(4):e12745. doi: 10.2196/12745 (PMC6538311; doi:10.2196/12745)
Supplement: Multimedia Appendix 2 [file mhealth_v7i4e12745_app2.pdf]

“Can’t Even Quit”

A pilot randomised, controlled trial of of an mHealth intervention to promote smoking abstinence for Aboriginal and Torres Strait Islander people

V2.0 03 Feb 2016

Contents

1. Administrative information ... 3
1.1 Trial Registration ... 3
1.2 Funding ... 3
1.3 Roles and Responsibilities ... 3
1.4 Glossary of abbreviations and terms ... 4
2. Protocol Synopsis ... 5
3. Introduction ... 7
3.1 Background ... 7
3.2 Intervention development ... 7
3.3 Objectives ... 8
3.4 Trial/study Design ... 8
4. Methods ... 8
4.1 Study Setting ... 8
4.2 Eligibility Criteria ... 8
4.3 Interventions ... 8
4.4 Outcomes ... 9
4.5 Participant timeline ... 9
4.6 Sample size ... 10
4.7 Recruitment ... 10
4.8 Allocation ... 10
4.9 Data collection methods ... 10
4.10 Data management ... 11
4.11 Statistical Methods ... 11
4.12 Data Monitoring ... 11
5. Ethics and Dissemination ... 11
5.1 Research Ethics Approval ... 11
5.2 Protocol Amendments ... 12
5.3 Consent ... 12
5.4 Confidentiality ... 12
5.5 Declaration of Interests ... 12
5.6 Access to data ... 13
5.7 Dissemination ... 13
6. References ... 14
7. Appendices ... 15
Appendix 1: Intervention features ... 15
Appendix 2: Project governance ... 21
1. Executive Committee terms of reference ... 22
2. Investigator Committee terms of reference ... 23
3. Working group terms of reference ... 24

1. Administrative information

1.1 Trial Registration

To be submitted to the Australian and New Zealand Clinical Trials Registry upon Human Research Ethics Committee approval

1.2 Funding

New South Wales Department of Health

1.3 Roles and Responsibilities

Investigator team
David Peiris (Principal Investigator)
Lachlan Wright
Jasmin Sarin
Jenny Hunt
David Thomas
Anthony Rodgers
Julie Redfern
Clara Chow

Contact for notices
Lachlan Wright
Project Manager, Cardiovascular Division, The George Institute for Global Health
Level 10, King George V Building
83-117 Missenden Rd Camperdown 2050 NSW
Tel: +61 2 999 34500
Fax: +61 2 999 34502

Committees
Three committees will guide the project- a project executive will oversee all aspects of the project, a working group will be responsible for the day-to-day operational activities, and an external advisory group will provide expert advice. An organisational diagram and terms of reference for each of these groups are attached in Appendix 1.

1.4 Glossary of abbreviations and terms

**ACCHS**	Aboriginal Community Controlled Health Service
**ACI**	Agency for Clinical Innovation
**AH&MRC**	NSW Aboriginal Health and Medical Research Council
**CAH**	Centre for Aboriginal Health, NSW Health
**CPH**	Centre for Population Health, NSW Health
**CI**	Cancer Institute NSW
**CQI**	Continuous Quality Improvement
**MSHR**	Menzies School of Health Research, Darwin
**TGI**	The George Institute for Global Health

2. Protocol Synopsis

Background

Although there have been recent encouraging declines in smoking cessation, in 2012-13, 41% of Aboriginal and Torres Strait Islander people over the age of 15 smoked cigarettes daily – over two times greater than the general population . The personal and societal impact of smoking-related diseases is inequitably distributed with, for example, Aboriginal and Torres Strait Islander peoples experiencing around five times greater cardiovascular disease burden than other Australians. Mobile health technologies are emerging as a cost-effective strategy to lower smoking rates but to date no such trials have been conducted in Australia.

Study design

A single blinded, pilot, randomised, controlled trial involving approximately 200 Aboriginal smokers with an average follow-up of 6 months.

Intervention

(1) A multifaceted smartphone application available on Android or iOS platforms that comprises a personalised profile and quit plan, behavioural strategies to increase motivation to quit and maintain an quit attempt, craving support, and a challenge feature in which users can ‘compete’ with others on a personalised challenge.
(2) Usual smoking cessation support provided through either the participating Aboriginal Community Controlled Health Service or the NSW Aboriginal Quitline.

Aims

We hypothesise that a multi-faceted intervention using a mobile phone application when combined with usual smoking cessation support services will double smoking abstinence rates at 6 months.

Patient eligibility

Consenting Aboriginal people (aged >16 years) with access to an Android or iOS phone who are willing to make a quit attempt in the next month.

Randomisation

Centrally performed web-based 1:1 allocation to the intervention versus usual smoking cessation services using a permuted block sequence stratified by study centre, gender and age (Figure 1)

Data collection

Data will be collected via telephone assessments at baseline, 4 weeks and 6 months by a project officer who is blinded to group allocation.

Primary Outcome

The primary outcome is self-reported continuous smoking abstinence, objectively verified at 6 months. Self-reported continuous abstinence is defined as no more than five cigarettes smoked in the past week at 4 weeks follow-up and no more than five cigarettes smoked since the start of the abstinence period at 6 months of follow-up. Self-reported smoking cessation will be confirmed with an Airmet Scientific Micro Plus Smokerlyzer (carbon monoxide metre breath test where a reading of >8 represents recent tobacco smoking). (10)

Secondary Outcomes

Secondary outcomes are point prevalence of abstinence (ie, no smoking in the past 7 days) at 4 weeks and 6 months, and self-reported continuous abstinence since the start of the abstinence period, 28-day abstinence, and use of other smoking cessation services (counselling, nicotine replacement therapy, medication) during the trial.

Feasibility and acceptability data will also be collected through surveys and usage analytics of the phone application.

Statistical power

Based on previous studies the abstinence rate is assumed to be double that of the control arm. 1000 participants would be needed to detect a 5% absolute difference (ie: 10.0% vs 5.0%; relative risk 2.00), assuming a 15% loss to follow-up, 2a=0.05 and 80% probability of detecting a significant difference with p values <0.05 judged as significant. This study will be a pilot RCT in which 200 participants will be recruited to assess feasibility, resource considerations, acceptability and preliminary effectiveness data to inform a future, adequately powered trial.

Using the secondary outcome measure of 7-day abstinence rates at 4 weeks, and assuming the control arm abstinence rate is 12% and the intervention arm abstinence rate is 2.4 times greater (as observed in previous studies), there will be 80% probability of detecting a significant difference with 200 participants from the pilot study.

Significance

There have been no controlled trials of mobile health strategies for smoking cessation in Australia and only one trial worldwide involving Indigenous populations. If the outcomes provide sufficient justification for a fully powered trial, it could make a major contribution on effective, scalable and cheap strategies to lower smoking related disease burden in Aboriginal communities.

Figure 1: Study Schema

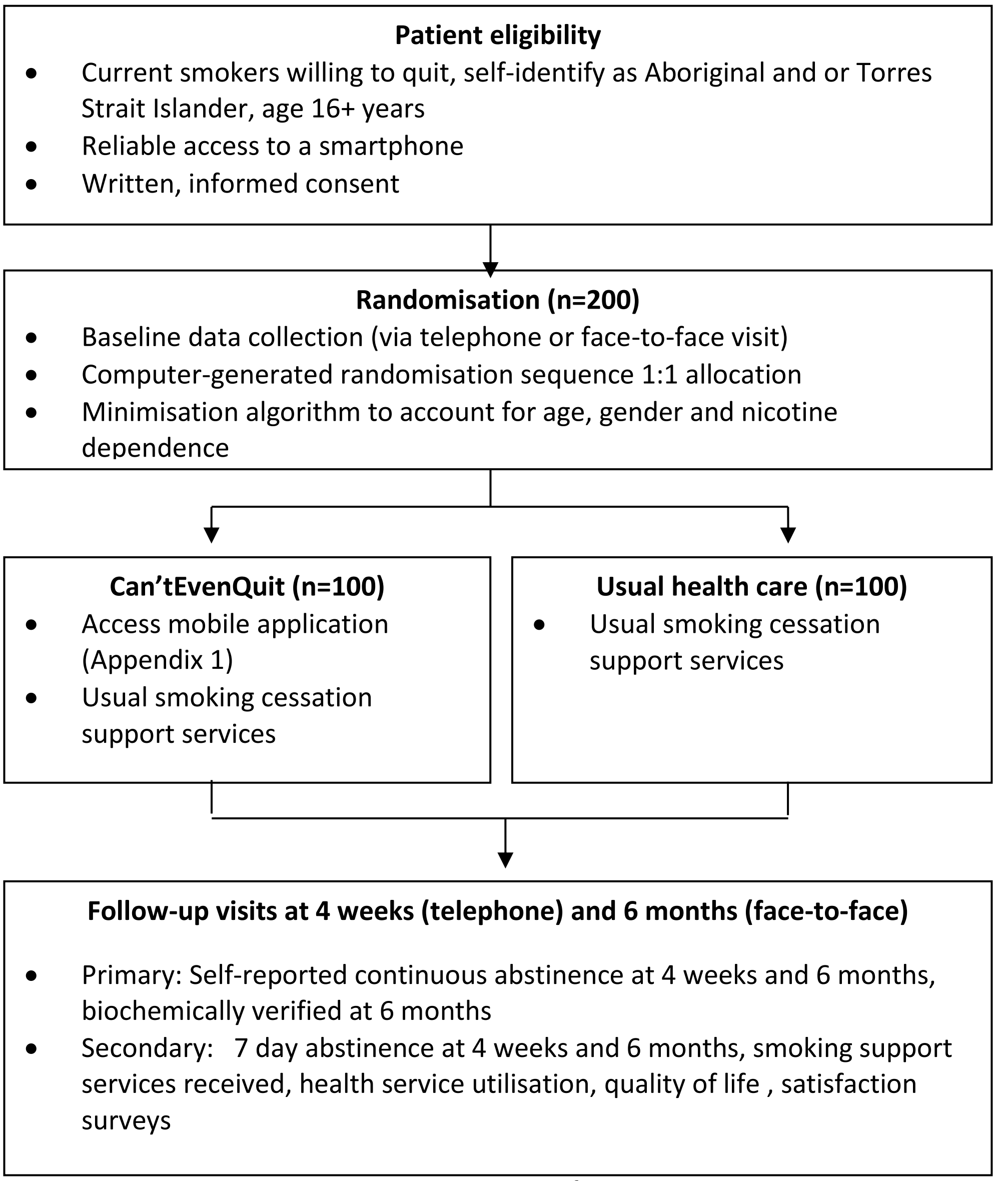

3. Introduction

3.1 Background

Despite Australia having one of the lowest rates of smoking in the world, Aboriginal and Torres Strait Islander people are more than twice as likely to smoke as other Australians. Although there have been recent encouraging declines in smoking cessation, in 2012-13, 41% of Aboriginal and Torres Strait Islander people over the age of 15 smoked cigarettes daily.(1) A recent national survey found that although Aboriginal and Torres Strait Islander smokers are less successful at quitting, they are more likely to have made a quit attempt in the last year than non-Aboriginal smokers (MJA in press). Smoking affects whole households with 62% of households with Aboriginal and Torres Strait Islander residents having a least one regular smoker.(2) The personal and societal impact of smoking-related diseases is inequitably distributed with, for example, Aboriginal and Torres Strait Islander peoples experiencing around five times greater CVD burden than other Australians.(3)

Mobile health (mHealth) is a term used for the practice of medicine and public health supported by mobile devices. It is a multidimensional field encompassing a wide variety of tools, technologies, and models of health care delivery. A systematic review of clinical trials to reduce smoking rates in Indigenous populations, conducted in 2013, analysed five studies- three testing Quitline protocols combined with cessation products compared with Quitline alone, and two using culturally adapted cessation counseling using mobile phones.(4) Outcomes were mixed, however, a New Zealand trial involving1705 participants (355 Maori) found a greater than two-fold increase in smoking abstinence at 6 weeks (28% vs. 13%) which appeared to be sustained at 6 months.(5) Importantly, abstinence rates were equally successful amongst Māori as non-Māori in the intervention arm.(6) This program is now being administered via NZ Quitline. The Txt2Stop trial adapted the NZ program for the United Kingdom. The study involving 5,800 participants across the UK, also demonstrated a doubling of quit rates over the control group at six months (verified abstinence of 10.7% v.4.9%).(7) The program has since been shown to be highly cost-effective and has been adopted by the National Health Service. A recent Cochrane systematic review found mobile phone-based smoking cessation interventions led to a 71% increased rate of smoking cessation when compared with control with the results largely driven by the outcomes in the NZ and UK trials.(8) There have not been any controlled trials of mHealth interventions for smoking cessation in Australia to date.

3.2 Intervention development

Our work on developing an mHealth intervention to assist Aboriginal and Torres Strait Islander smokers in abstaining from smoking was conducted in three phases:

1. Landscape analysis: A review of existing smoking cessation support services, products and research involving Aboriginal and Torres Strait Islander people with a specific focus on people living in NSW was conducted. The ATRAC Framework – A Strategic Framework for Aboriginal Tobacco Resistance and Control in NSW, which forms the blueprint for policy in NSW was also reviewed. In parallel with this review a competitor analysis of national and international mobile applications that have been used for smoking cessation was conducted.

2. User centred app design: An expert user group involving current smokers and ex-smokers from Aboriginal Community Controlled Health Services was convened to guide intervention development. A series of interactive sessions were conducted with this user group in which user experience, attitudes toward existing smoking cessation services and mobile applications were canvassed. From this a series of hypothetical user personas were developed. This enabled a deep contextual understanding of user experiences, knowledge, attitudes, behaviour related to both tobacco use and

use of mobile technology, triggers for behaviour change and past experiences with quitting and cessation maintenance. Following this, a series of rapid visualisation design cycles were conducted to develop and refine app development until the final software specification was agreed on.

In parallel with this process the existing bank of motivational text messages was adapted in consultation with user groups to ensure they were appropriate for use in this setting.

3. Software development: Once the software specification was finalised the development phase commenced. An existing text-message bank which has been used in previous studies was adapted following previously used methodology to enhance applicability and acceptability to the target population.(9) This involved user feedback and focus group testing of these with Aboriginal community members in collaboration with existing ACCHS partners. The existing text messages were modified accordingly and a series of new messages were added to the database.

3.3 Objectives

The overall project goal is to develop and evaluate an mHealth intervention to assist Aboriginal people in NSW with smoking cessation assistance. We hypothesise that a multi-faceted smoking cessation mobile application (Can'tEvenQuit), provided as part of routine smoking cessation services, will result in a doubling of biochemically verified smoking abstinence rates at 6 months when compared with usual care.

3.4 Trial/study Design

The Can't Even Quit trial will be evaluated using a single-blind randomised controlled trial involving 200 regular health service attendees who are current tobacco smokers

4. Methods

4.1 Study Setting

Three study settings will be used to recruit interested participants:

- Aboriginal Community Controlled Health Services
- Aboriginal Quitline
- Agency for Clinical Innovation – 1 Deadly Step program

4.2 Eligibility Criteria

Participants will be eligible if they are able to give informed consent and meet all of the following criteria:

- Current smokers aged 16 years or older
- Self-identification as an Aboriginal and/or Torres Strait Islander person
- Willing to make an attempt to quit smoking in the next month
- Access to an iPhone or Android smartphone

4.3 Intervention arm

All participants will be free to participate in any other smoking cessation service or support that they wish to use, and will be offered Quitline and local ACCHS contact numbers. Participants allocated to the intervention group will have the opportunity to download and register using the Can'tEvenQuit app immediately. The app will be accessible via the Apple App Store and Google Play. Intervention

arm participants will be able to invite external people to use a simplified version of the app to support participation in a quit smoking challenge.

A support worker will facilitate with registration profile, password set-up and establishing a tailored quit plan. Participants will then be given a tutorial of app feature including how to set up a challenge, motivational support and tracking progress on their plan. App set up can also be done without the use of a support worker if desired. Detailed information about the intervention content, design and features are provided in Appendix 1.

4.4 Control arm

Participants in the control arm will continue with their usual health care. They will be encouraged to take advantage of all available smoking cessation support services offered either through the ACCHS or through Quitline. Although participants in the control group could potentially download the app, they will be unable to successfully register to use it (via a database cross-check). At the end of 6 months follow-up control arm participants will be offered access to the app should they be interested in using it for a minimum 6-month period.

4.4 Outcomes

The primary outcome is self-reported continuous smoking abstinence, objectively verified at 6 months. Self-reported continuous abstinence is defined as no more than five cigarettes smoked in the past week at 4 weeks follow-up and no more than five cigarettes smoked since the start of the abstinence period at 6 months of follow-up. Self-reported smoking cessation will be confirmed with an Airmet Scientific Micro Plus Smokerlyzer (carbon monoxide metre breath test where a reading of >8 represents recent tobacco smoking). (10)

Secondary outcomes are point prevalence of abstinence (ie, no smoking in the past 7 days) at 4 weeks and 6 months, and self-reported continuous abstinence since the start of the abstinence period, 28-day abstinence, and use of other smoking cessation services (counselling, nicotine replacement therapy, medication) during the trial.

4.5 Participant timeline

**Assessment Description**	**Screening**	**Baseline/ Randomisation/ Enrolment**	**Visit 1 Telephone (4 weeks)**	**Final visit Face-to-face (6 months)**
Informed Consent	X			
Eligibility	X	X		
Reasons for non-participation	X	X		
Demographics, Medical History, Medications		X	x	x
Questionnaires - Quality of Life, use of health services		X	X	X
Carbon monoxide metre breath test				X
Serious Adverse Events		X	X	X

4.6 Sample size

On the basis of the Text2Quit and Text2Stop trials we estimate the control arm abstinence rate at 6 months to be 5%. Assuming that the abstinence rate is double that of the control arm, we calculate that 1000 participants would be needed to detect a 5% absolute difference (ie: 10.0% vs 5.0%; relative risk 2.00), assuming a 15% loss to follow-up, 2a=0.05 and 80% probability of detecting a significant difference with p values <0.05 judged as significant. This study will be a pilot RCT in which 200 participants will be recruited to assess feasibility, resource considerations, acceptability and preliminary effectiveness data to inform a future, adequately powered trial.

Using the secondary outcome measure of 7-day abstinence rates at 4 weeks, and assuming the control arm abstinence rate is 12% and the intervention arm abstinence rate is 2.4 times greater (as observed in the Text2Stop trial), there will be 80% probability of detecting a significant difference with 200 participants from the pilot study under the same assumptions as above.

4.7 Recruitment

Recruitment will take place via three settings. Up to four Aboriginal Community Controlled Health Services will be invited to participate in the study. Callers to the Aboriginal Quitline and community members screened at a 1 Deadly Step event will also be invited to participate. Interested individuals will be contacted by the project manager to discuss participation.

4.8 Allocation

Randomisation will be single blinded and conducted through the George Institute's central, computer-based randomisation service. Allocation will be 1:1 intervention versus control using a minimisation algorithm to valance for sex, age and Fagerstrom score for nicotine dependence. Staff involved in data collection and analysis will be blinded to treatment allocation.

4.9 Data collection methods

Eligibility assessment will be done in collaboration with the participating sites. All study instruments are administered by questionnaire (with the exception of the carbon monoxide assessment at the end of study.) Baseline data collection, consent and randomisation will be conducted by telephone or via a face to face visit with a trained project officer from the George Institute. For participants allocated to receive the Can'tEvenQuit app, a text message will be sent to the participant's phone with instructions on how to download the app. Participants will then have the option of a self-guided assessment to setting up the app or will be invited to meet with a research staff member to have a face-to-face visit to assist with app installation and set-up. All participants allocated to the intervention will be telephoned at 1 week to determine if they need any support with using the app. There will also be a "contact us" number in the app itself to call for help. At 4 weeks an "evaluation" project officer who is blinded to allocation status will conduct an outcome assessment by telephone for all participants in the trial. At 6 months post randomisation, this officer will conduct the final outcome assessment and biochemical verification of smoking status as part of a face-to-face visit.

In addition to the formal outcome assessment visits an analytic tool built in the app will assess usage patterns, which app features are used and any changes in usage patterns over time. A clinic record audit of smoking cessation support services will also be conducted for consenting participants recruited through ACCHSs.

An automated de-identified data extraction tool will also be provided to participating ACCHSs to assess smoking prevalence, recording rates and provision of smoking cessation assistance at the beginning and end of study for the entire regular client population to assess any changes during the trial period based on methods used previously.(11)

4.10 Data management

Non-electronic study data will be stored securely at The George Institute with access restricted to the principal investigators and the data management staff member responsible for maintenance of the database. Signed participant consent forms will be stored in the Investigator Site Folder at the participating health service. At the completion of the trial the consent forms will be brought back to The George Institute to be filed with essential study documents for 15 years. The investigator shall retain and preserve one copy of all data generated in the course of the study, specifically including but not limited to those documents defined by Good Clinical Practice as essential documents, for 15 years following study closure.

4.11 Statistical Methods

Intention-to-treat principle will be followed, and characteristics will be compared between the groups at 4 weeks and 6-month follow-up using χ^2 tests for categorical variables and t-tests for continuous variables. Results will be reported in terms of relative risks, 95% CIs and two-sided p values. Analyses will be undertaken using SAS Enterprise Guide (version 5.0). For the pilot study no sub-group analyses will be pre-specified.

4.12 Data Monitoring

The study will be monitored by a representative of The George Institute for Global Health. Site monitoring visits will be performed periodically, and communication by telephone, mail and e-mail will be used as needed to supplement site visits. The investigator and study personnel will assist the monitoring staff by providing all appropriate documentation, and being available to discuss the study.

Serious adverse events (SAE) will be collected at each of the two study visits. An SAE is any adverse event (any untoward, undesired, unplanned clinical event in the form of signs, symptoms, disease, or laboratory or physiological observations occurring in a human being participating in a clinical study, regardless of causal relationship) that (1) results in death; (2) is life threatening; (3) requires hospitalization or prolongation of an existing hospitalization; (4) results in a persistent or significant disability or incapacity. Given this is a pilot study a Data Monitoring Committee will not be needed and no interim analyses will be performed.

5. Ethics and Dissemination

5.1 Research Ethics Approval

The study will be conducted in accordance with the principles set out in the Declaration of Helsinki and its subsequent amendments of Tokyo, Venice, Hong Kong and Somerset West and the National Statement on Ethical Conduct in Human Research (2007), National Health and Medical Research Council Values and Ethics: Guidelines for Ethical Conduct in Aboriginal and Torres Strait Islander Health Research and the NSW Aboriginal Health Information guidelines.

An application requesting approval to conduct this study will be submitted to the Aboriginal Health & Medical Research Council Human Research Ethics Committee (HREC). Formal approvals from each of the participating sites will also be sought. Specific effort will be taken to respect the autonomy and governance of participating ACCHSs. It is also recognised that ACCHSs have rights and responsibilities regarding the use of health-related information for their attending clients. This includes the right to protect and care for the use of this information, the right to determine culturally appropriate forms of its reporting, and the right to grant or withhold permission from other agencies or organisations for its use. The content and format of any flyers, invitation letters, patient information statements and consent forms will be approved by the HREC and formatted in accordance with their own guidelines and requirements.

The study will not commence in any centre until all the necessary documentation has been completed. The Principal Investigator will be responsible for producing regular status reports, serious adverse event reports, and any other required documentation to the relevant HRECs in accordance with their guidelines. Any amendments or additions to the study protocol and material will be notified to the HREC by the Principal Investigator. It is the responsibility of the Principal Investigator to maintain up to date records of all correspondence and applicable documentation with the relevant HRECs and the regulatory authorities. The template of the Informed Consent Forms and Patient Information Statements, together with a copy of all signed Informed Consent Forms and any other consent related correspondence will also be kept in a separate file for audit purposes. All study records and documents will be stored for a minimum of 15 years from the end of the study or for a period as required by the HREC.

5.2 Protocol Amendments

Any significant change in the study protocol will require an amendment. Once the study Management Committee has approved a protocol amendment, the principal investigator will submit this to each HREC for written approval. The approval letter, signed by the HREC chair, must refer specifically to the investigator, the protocol number, the protocol title, the protocol amendment number, and the date of the protocol amendment. The protocol amendment may be implemented only after it has been approved by the HREC. A protocol change intended to eliminate an apparent immediate hazard to subjects may be implemented immediately, but the change must then be documented in an amendment, reported to the HREC and the study Management Committee within five working days.

If the revision is an administrative change (such as the addition or removal of committee members), a letter explaining the change(s) and a copy of the amended pages(s) in the protocol will be submitted to the HREC for its records. No formal approval from the HREC is required prior to implementation of administrative changes.

If any investigator retires, relocates, or otherwise withdraws from conducting a study, the responsibility for maintaining records may be transferred to The George Institute for Global Health, HREC, or other investigator. The George Institute for Global Health must be notified of and agree to the change. All associated documentation must also be updated.

5.3 Consent

The Project Officer should fully inform the potential participant of all aspects of the trial. Before consent may be obtained the Project Officer should give the participant time and the opportunity to ask questions. Verbal telephone consent must be obtained from the participant before any study related procedure is conducted. Documentation of consent will be filed electronically in a dedicated, web-based case report form. The patient may withdraw consent to participate in the study at any time during the study. If a patient withdraws consent, access to the app will discontinue and the participant can pursue any assistance for smoking cessation as needed. In this situation, specific consent will be sought to still conduct an end of study visit and to use any study data collected.

5.4 Confidentiality

All information about potential and enrolled participants will be collected electronically on secure, password protected, case report forms hosted on a study website by the George Institute. Only authorised George Institute staff will have access to participant data. Reports provided to the funder and to participating sites will not identify any individual and will contained aggregated data only.

5.5 Declaration of Interests

Financial and other competing interests for committee and investigators for the overall trial and at each study site will be collected and declared.

5.6 Access to data

Authorised personnel at the George Institute will have access to the final trial dataset. Any data sharing with other parties will be formulated in contractual agreements.

5.7 Dissemination

Interim and final project evaluation reports will be provided to the funder. Investigators will be encouraged to disseminate information from the project in a manner that supports health improvement for Aboriginal peoples and local benefit to participating ACCHSs. Collaborators will be required to seek written consent from ACCHSs to publicly disclose information that has been collected as a result of participation in the study. Publications arising from this study will require approval from the HREC prior to publication. The funder will be acknowledged in any publications arising from the study.

6. References

1. Australian Bureau of Statistics. Australian Aboriginal and Torres Strait Islander Health Survey: First Results, Australia, 2012-13 Cat series no: 4727.0.55.001 Canberra: ABS, 2014.
2. Australian Bureau of Statistics. Tobacco Smoking - Aboriginal and Torres Strait Islander people: A snapshot, 2004-05 Cat series no: 4722.0.55.004 Canberra: ABS, 2007.
3. Theo Vos BB, Lucy Stanley and Alan D Lopez. The burden of disease and injury in Aboriginal and Torres Strait Islander peoples 2003. Brisbane: School of Population Health, The University of Queensland; 2007.
4. Johnston V, Westphal DW, Glover M, Thomas DP, Segan C, Walker N. Reducing Smoking Among Indigenous Populations: New Evidence From a Review of Trials. Nicotine & Tobacco Research. 2013;15(8):1329-38.
5. Rodgers A, Corbett T, Bramley D, Riddell T, Wills M, Lin R-B, et al. Do u smoke after txt? Results of a randomised trial of smoking cessation using mobile phone text messaging. Tobacco Control. 2005;14(4):255-61.
6. Bramley D RT, Whittaker R, Corbett T, Lin R-B, Wills M, Jones M, Rodgers A,. Smoking cessation using mobile phone text messaging is as effective in Maori as non-Maori. . New Zealand Medical Journal 2005;118(1216):U1494.
7. Free C, Knight R, Robertson S, Whittaker R, Edwards P, Zhou W, et al. Smoking cessation support delivered via mobile phone text messaging (txt2stop): a single-blind, randomised trial. The Lancet.378(9785):49-55.
8. Whittaker Robyn MH, Bullen Chris, Borland Ron, Rodgers Anthony, Gu Yulong. Mobile phone-based interventions for smoking cessation Cochrane Database of Systematic Reviews. 2012;11(10.1002/14651858.CD006611.pub3).
9. Chow CK, Redfern J, Thiagalingam A, Jan S, Whittaker R, Hackett M, et al. Design and rationale of the tobacco, exercise and diet messages (TEXT ME) trial of a text message-based intervention for ongoing prevention of cardiovascular disease in people with coronary disease: a randomised controlled trial protocol. BMJ Open. 2012;2(1).
10. Cunnington AJ, Hormbrey P. Breath analysis to detect recent exposure to carbon monoxide. Postgraduate Medical Journal. 2002;78(918):233-7.
11. Peiris D, Usherwood T, Panaretto K, Harris M, Hunt J, Redfern J, et al. Effect of a Computer-Guided, Quality Improvement Program for Cardiovascular Disease Risk Management in Primary Health Care: The Treatment of Cardiovascular Risk Using Electronic Decision Support Cluster-Randomized Trial. Circulation: Cardiovascular Quality and Outcomes. 2015;8(1):87-95.

7. Appendices

Appendix 1: Intervention features

There are five core elements to the mobile application:

1. Log in/ Profile set up/ Home screen
2. My Quit Plan
3. Motivate Me
4. My Cravings
5. My Challenges

1. Log In/Set Your Profile/ Dashboard

- Participants will be provided with instructions on how to download the app and follow a standard registration process to log in
- Once logged in they will be provided with simple steps to set up their profile including personalising their profile with photos etc
- Once set up they will be taken to a home screen where they can then explore other features of the app
- This set up process may be facilitated by a trained Aboriginal Health Worker or may be conducted through self-registration

Figure 1: Registration set-up screenshots

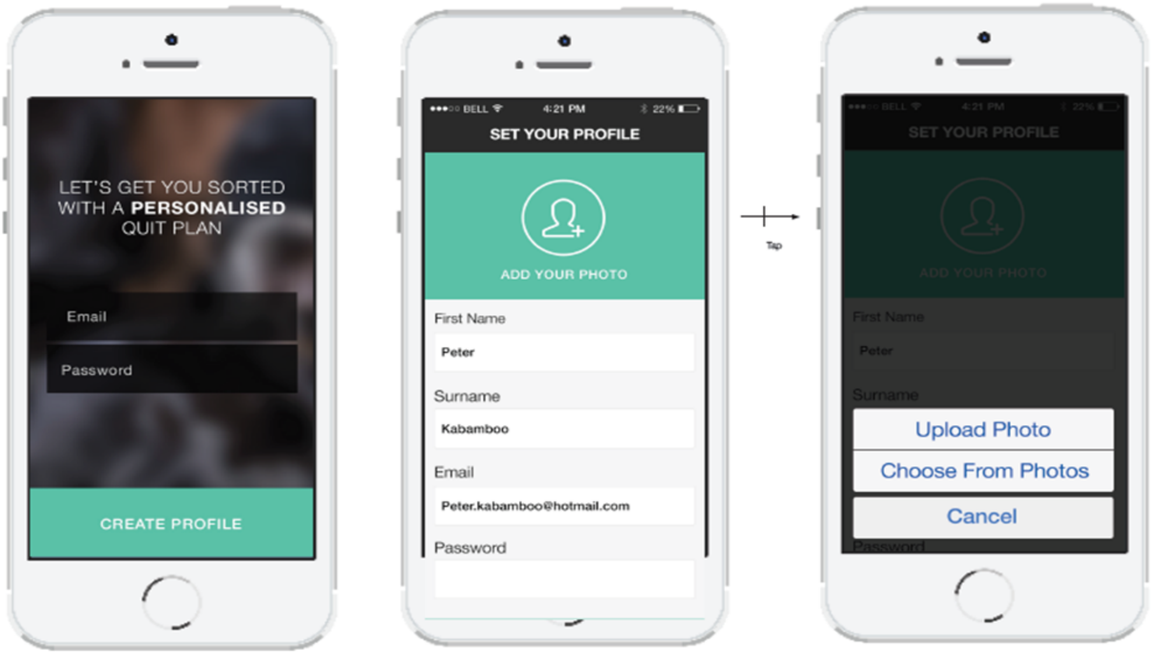

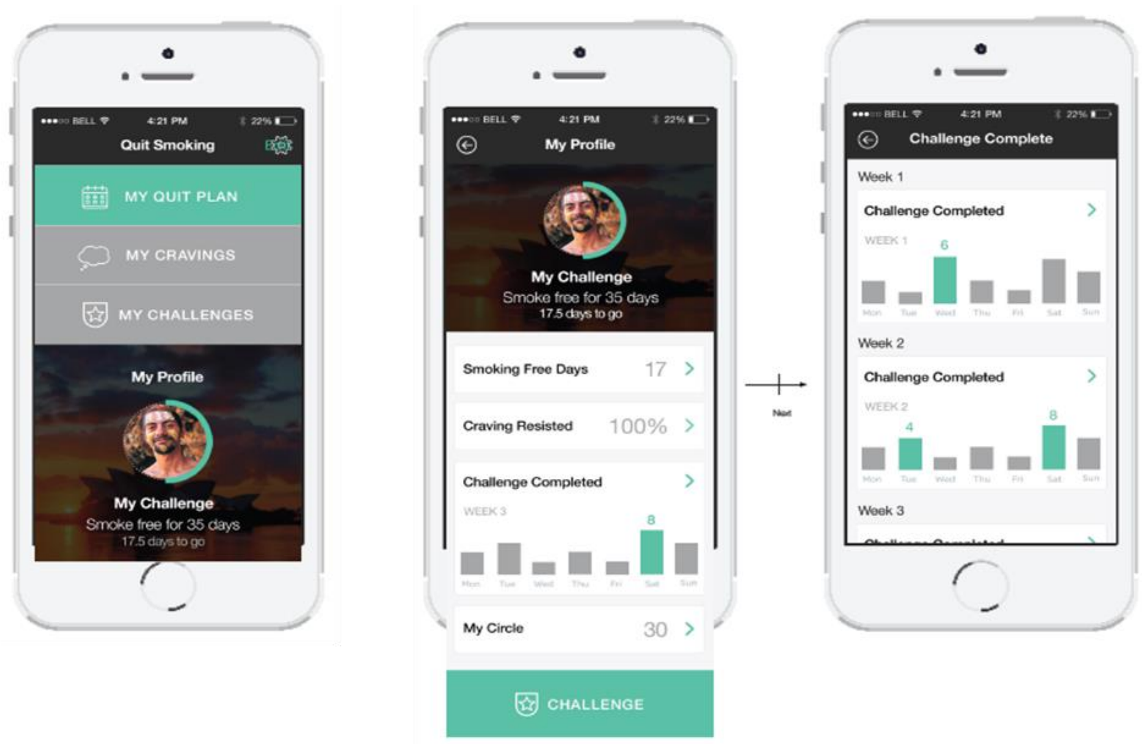

2. My Quit Plan

The core features of My Quit Plan are:

- An option to set it up straight away or later down the track
- A series of questions about smoking habits and current lifestyle
- Information regarding past quit attempts
- Why, when and how the user would like to quit or how they would like to maintain quitting
- This information will then be used to create a personalised quit-plan
- The quit plan will then provide a visual graphic of their journey to quitting combined with information on health and other benefits at different time points post quitting (eg money saved, return of lung function) This will also feature in the 'motivate me' section below
- Information on Nicotine Replacement Therapy and medications to assist with quit attempts including a medication adherence tracker

Figure 2: My quit plan screenshots

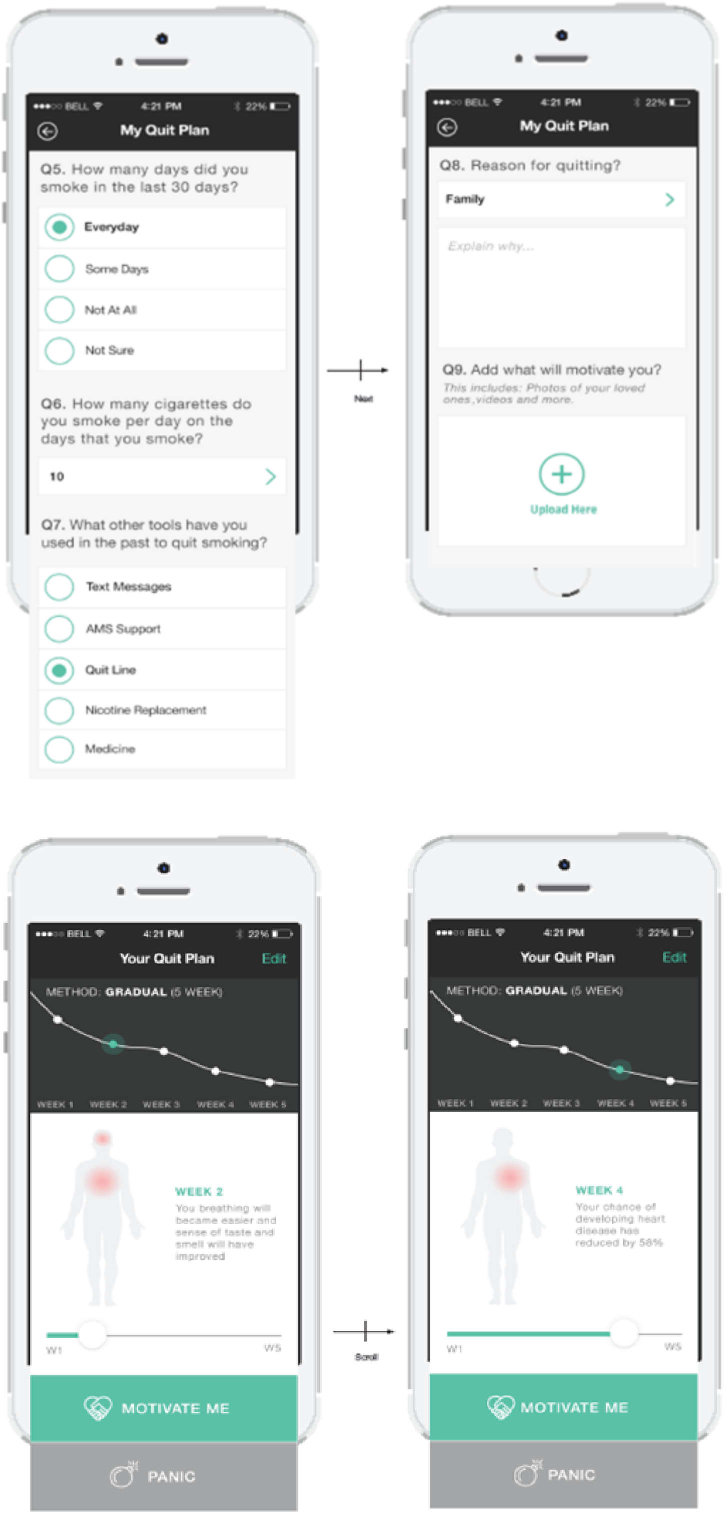

3. Motivate Me

The main features of this function include:

- The ability to upload multiple photos or videos to provide participants with emotionally meaningful stimuli to remain abstinent from smoking
- A motivational SMS message bank feature. Similar to the Text2Quit and Text2Stop trials, this bank of messages consists of several hundred messages delivered at random times of the week. There will also be a ‘crave’ function where if the user activates a panic button and a rapid sequence of messages will be sent to help overcome an acute craving period. Message content has been tailored following consultation with Aboriginal community user groups
- No-smoking multimedia advertisements
- Health tips (related to the quit plan)

Figure 3: Motivate me screenshots

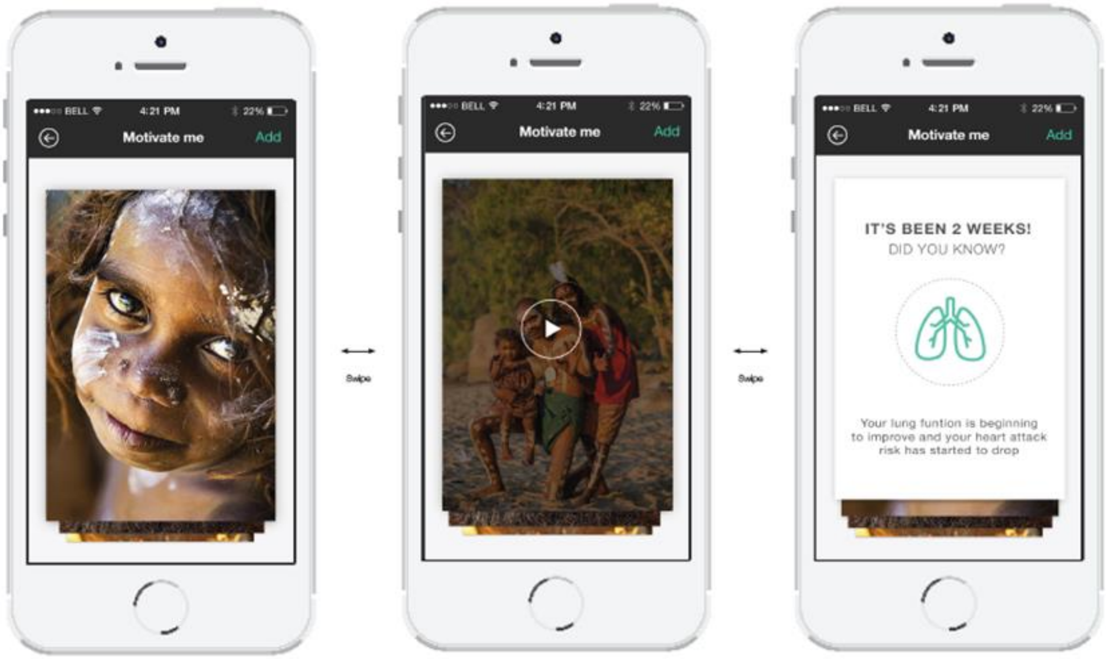

4. My Cravings

- A call function to enlist support from a buddy or someone else
- Receipt of a higher frequency of SMS motivational messages
- A craving tracker to calculate the number of cravings and the number resisted
- This will inform the app and adjust the personalised quit plan with respect to the frequency of supportive messaging, interactions with AMS health workers or suggestions on ways to improve your mood

Figure 4: My cravings screenshots

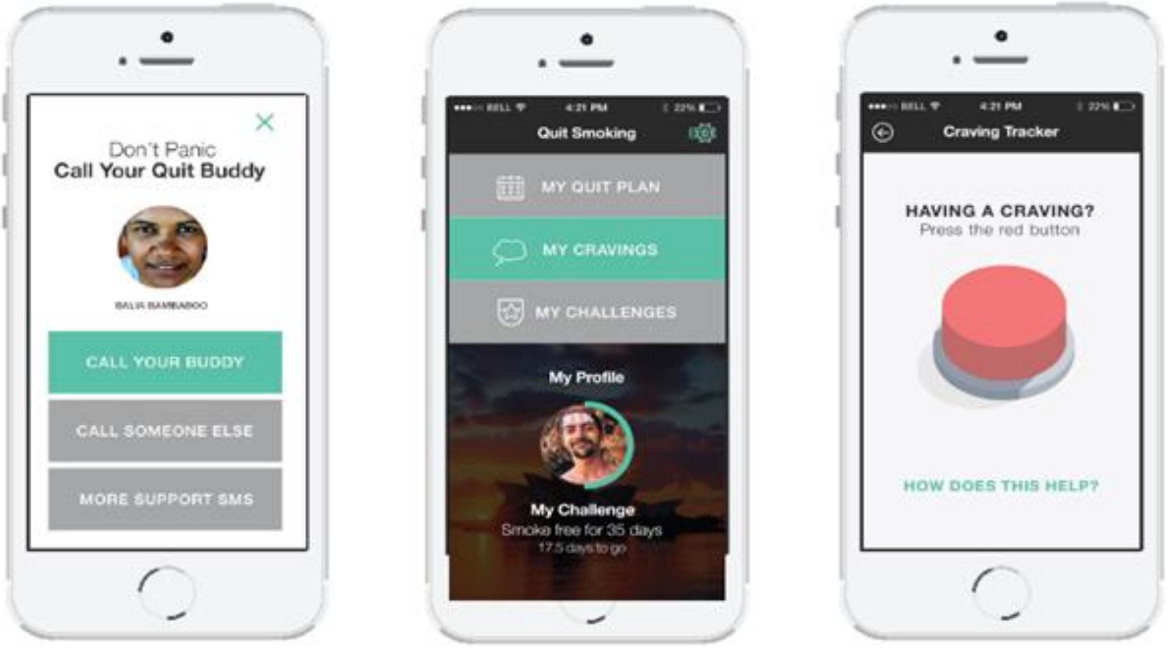

5. My Challenges

- Challenge others and compete. A simple workflow has been designed to enable others to join a challenge.
- Participants will be able to drag and drop people into their “circle” to invite them to a challenge. They can set the challenge type, date and duration.
- Receivers of a challenge will receive a pop-up or push notification or SMS message inviting them to the challenge. They will be able to view who has challenged them, view others who are taking part and decide to participate now or next time. For new participants instructions will be given on how to set up the app to participate in the challenge. A challenge can be issued to anyone, however, participants in the control arm of the trial will be prevented from being invited into a challenge.
- Once a challenge is set participants will be able to view the progress of others attempting the same challenge, whether they “craved and caved” on the challenge and view and share their progress with others via standard social media platforms like Facebook.

Figure 5: My Challenges screenshots

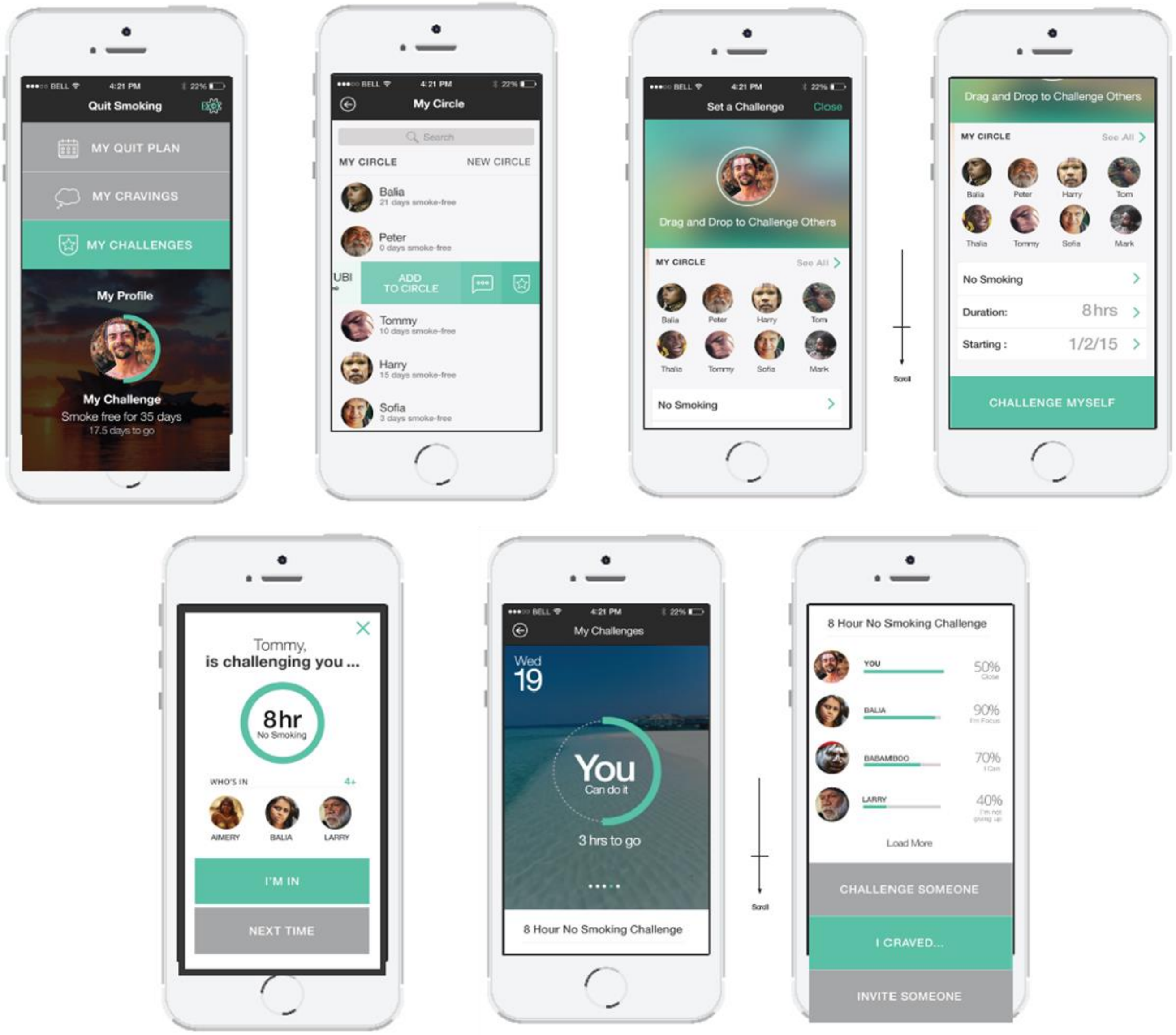

Appendix 2: Project governance

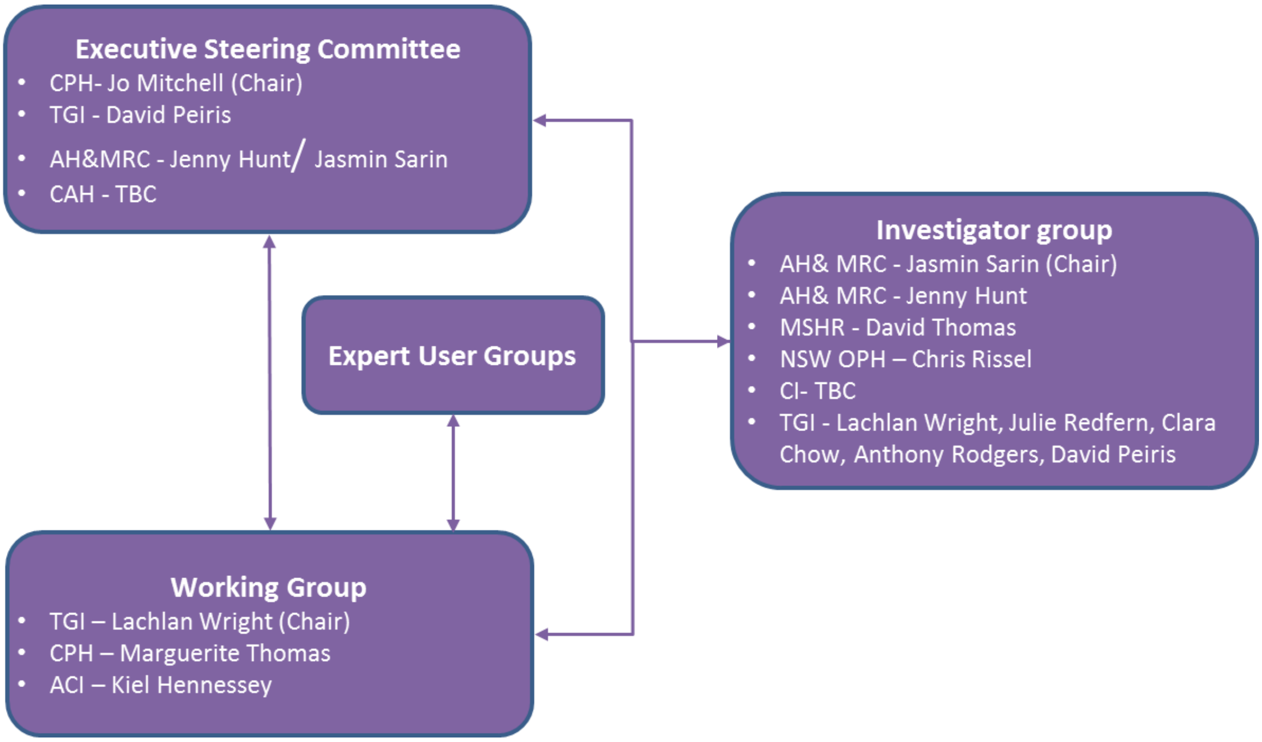

- CPH- Centre for Population Health, NSW Health
- CAH- Centre for Aboriginal Health, NSW Health
- AH&MRC Aboriginal Health and Medical Research Council
- ACI- Agency for Clinical Innovation
- MSHR – Menzies School of Health Research
- CI- Cancer Institute
- NSW OPH- NSW Office of Preventive Health
- TGI- The George Institute

1. Executive Committee terms of reference

Membership

- NSW Ministry of Health:
    - Centre for Population Health
    - Centre for Aboriginal Health
- The George Institute for Global Health
- Aboriginal Health & Medical Research Council (AH&MRC)

Terms of reference

The Executive Committee will oversee project delivery. The Executive Committee will report to the NSW Aboriginal Health Partnership Committee on Tobacco Resistance and Control.

Chair

The Executive Committee will be chaired by Dr Jo Mitchell, Director, Centre for Population Health.

Meetings

The Executive Committee will meet quarterly, with more frequent meetings in the set up phase. The Executive Committee meetings will be conducted face-to-face, with participation via teleconference available to those unable to attend in person. Out of session correspondence will be by email.

Secretariat

The Centre for Population Health, NSW Health will provide Secretariat support.

Agenda

The agenda will be set by the Chair and will be circulated to Executive Committee members prior to each meeting.

Minutes

The minutes are to be disseminated within two weeks of each meeting by the Secretariat. Minutes will be forwarded to Executive Committee members.

Life of the Executive Committee

The life of the Executive Committee will be from June 2014 to December 2016 in line with the project schedule outlined in the Agreement between the Health Administration Corporation and the George Institute. However, a review of group membership for both groups will occur in 2015 to ensure that membership remains relevant.

2. Investigator Committee terms of reference

Proposed membership

- AH&MRC
- Menzies School of Health Research
- Cancer Institute NSW
- The George Institute

Terms of reference

The Investigator Committee will provide oversight on all scientific aspects of the trial. It will report to the Executive Committee.

Chair

The Investigator Committee will be chaired by Jasmin Sarin, Aboriginal Health & Medical Research Council.

Meetings

The Investigator Committee will meet quarterly. Meetings will be conducted face-to-face, with participation via teleconference available to those unable to attend in person. Out of session correspondence will be by email.

Secretariat

The George Institute will provide Secretariat support

Agenda

The agenda will be set by the Chair and circulated to group members prior to each meeting.

Minutes

The minutes are to be disseminated within two weeks of each meeting by the Secretariat. Minutes will be forwarded to committee members.

Life of the Investigator Committee

Investigator Committee will be from April 2015 to December 2016.

3. Working group terms of reference

Proposed membership

- Centre for Population Health, NSW Ministry of Health
- The George Institute
- Agency for Clinical Innovation (as required by the project)

Terms of Reference

The key role of the working group is day to day management of the project. The working group will report to the Executive Committee

Chair

The Working Group will be chaired by Lachlan Wright, The George Institute.

Meetings

It is planned that the Working Group will meet on a fortnightly basis. The Working Group will be conducted face-to-face, with participation via teleconference available to those unable to attend in person. Out of session correspondence will be by email.

Secretariat

The George Institute will provide Secretariat support.

Agenda

The agenda for Working Group meetings will be set by the Chair and will be circulated to group members prior to each meeting.

Minutes

The minutes are to be disseminated within two weeks of each meeting by the Secretariat. Minutes will be forwarded to the Working Group members

Life of the Working Group

The life of the Working Group will be from June 2014 to December 2016 in line with the project schedule outlined in the Agreement between the Health Administration Corporation and the George Institute. However, a review of group membership will occur in 2015 to ensure that membership remains relevant.
