# Supplementary material for: A Smartphone App to Assist Smoking Cessation Among Aboriginal Australians: Findings From a Pilot Randomized Controlled Trial
Source: JMIR Mhealth Uhealth. 2019 Apr 2;7(4):e12745. doi: 10.2196/12745 (PMC6538311; doi:10.2196/12745)
Supplement: Multimedia Appendix 3 [file mhealth_v7i4e12745_app3.pdf]

Multimedia Appendix 3

Development of the mHealth intervention was conducted in three phases:

Landscape analysis

A competitor analysis of national and international mobile applications that have been used for smoking cessation was conducted. Table 1 shows the top nine apps we identified in the review and their key features. There was a high degree of concordance in application features such as the ability to track progress after quitting, money saving calculators, motivational tools to deal with cravings and setback, reward functions and social engagement tools.

Table 1: Top rated apps on landscaping analysis

**App**	**Country**	**Platform**	**User score (max 5)**	**Features**
My Quit Buddy	Australia	iOS & Android	Google Play 3.8<br>App Store 4	Tracks number of cigarettes avoided, money saved, nominate "danger times", "distract me" feature, set goals, and "buddy up" with friends and family for extra encouragement
Livestrong MyQuit Coach	USA	iOS & Android	Google Play 2.8<br>App Store 4.4	Reviewed by doctors, virtual coach, personalized plan for quitting, tracks smoking and nicotine cravings, sets reminders, goals and personal motivations, achievement badges as a reward for progress, and can communicate with other users via the app's built-in social support circle.
Quit it Lite	Germany	iOS	App Store 4.5	Feedback on amount of tar not consumed, money-saving goal
Quit Smoking: Cessation Nation	USA	Android	Google Play 4.5	Tracks health improvements, money saved, and provides reward badges for progress; includes a game to help distract from your cravings.
Smoke Free	UK	Android & iOS	App Store 5<br>Google Play 4.6	Tracks money saved, period smoke free, number of cigarettes not smoked, amount of life regained and health improvements.

App	Country	Platform	User score (max 5)	Features
				‘Missions’ to support quit attempts, craving log function with motivational tips to deal with them, a map tracker to see where cravings are greatest, make notes to see what patterns can be identified.
Quit for you- Quit for two	Australia	Android & iOS	Google Play 3.7 App Store 4.5	Provides practical quit tips and advice for pregnant women: quit tips on dealing with cravings, games to distract from cravings, inspiring daily updates, baby name selector, breathing yoga exerciser, week-by-week facts about baby's development, daily savings announcements based on money not spent on smoking
QuitNow	USA	Android & iOS	Google Play 4.5 App Store 4	Feedback data on the time since the last cigarette, number of cigarettes have avoided, money saved. Provides indicators on health improvement process, an achievement list which is unlocked as time quit time lengthens. Social elements to share achievements, chat with other quitters, get help, tips and tricks from others. Integrates with Facebook, Twitter, and other Social Networks
Free Butt Out	Canada	Android & iOS	Google Play 3.4 App Store 4.5	Craving logger and tailored quit plan generator
Kwit	France	iOS & Android	Google Play 4.5 App Store 4.5	Uses of game design techniques, game thinking and game mechanics, provides information on time stopped smoking, money saved and the number of cigarettes not smoked, 60 achievements progressively unlocked as quit time lengthens, integration with Facebook, Twitter, uses accelerometer in the phone and once shaken a motivational card from a list of 40 different cards to manage cravings.

We found only one published review from New Zealand that ranked smartphone apps for smoking cessation by their quality and assessed their appropriateness for use by Māori communities. Apps were assessed for their 'Mobile Application Rating Scale' which assesses the apps for engagement, functionality, aesthetics, information and subjective quality. Each app was also assessed against smoking cessation criteria and their level of Māori-specific cultural appropriateness. This review found that the highest scoring app was "Quit Now: My QuitBuddy" which was produced by the Australian National Preventive Health Agency. Despite this being the highest ranking app, the review concluded that even the highest scoring apps did not perform particularly well against the smoking cessation specific criteria or the cultural appropriateness criteria as a whole.

User centred app design

An expert user group involving current smokers and ex-smokers from Aboriginal Community Controlled Health Services was convened to guide intervention development. A series of interactive sessions were conducted with this user group in which user experience, attitudes toward existing smoking cessation services and mobile applications were canvassed. In collaboration with the software developer, a series of hypothetical user personas were developed. This enabled a deep contextual understanding of user experiences, knowledge, attitudes, behaviour related to both tobacco use and use of mobile technology, triggers for behaviour change and past experiences with quitting and cessation maintenance. Following this, a series of rapid visualisation design cycles were conducted to develop and refine app development until the final software specification was agreed on.

The app name 'Cant Even Quit'

'Can't Even Quit' was derived from a popular sketch on *Black Comedy*, an ABC comedy show created by, written by, and starring Aboriginal people. The Can't Even.. sketch involves two Aboriginal men challenging each other by saying Can't Even Dance, Can't Even Sing, Can't Even Shop etc. They then proceed to prove to the other that they can do it better. A major feature of the app and differentiating feature from other smoking apps is the 'My Challenges' function. The idea for this function arose from consultations with the expert Aboriginal user group and given the popularity of Black Comedy, there was strong interest in naming the app "Can't Even Quit." Although for people not familiar with Black Comedy the app name may convey negative connotations, the response from Aboriginal community members was quite different as it conveyed a sense of fun to approaching smoking cessation.

Text message bank development

In parallel with this process, we were provided with an existing text message bank used by Quit Victoria as part of the QuitTxt intervention. It provides tailored messages focussed around the participant's quit attempt date. Messages are delivered to assist with preparation in the lead up to the quit attempt, messages on quit day, the immediate period post quitting, and maintenance messages for up to 6 months' post-

quitting. Users could select a level of message frequency of low, medium or high in the app.

Message frequency per day according to intensity of use and stage of quitting

	**Intensity**		
**Quit stage**	**High**	**Medium**	**Low**
Quit date > 2 days away	4	3	2
Quit date 2 days away	6	4	3
Quit date tomorrow	8	6	4
Quit day	8	6	4
D1-2 post quit	8	6	4
D3-7 post quit	6	4	3
D8-14 post quit	4	3	2
D15-30 post quit	3	2	1
>D30 post quit	7	4	2
**Emergency messages**	Single burst of 2 messages 30 minutes apart		

Additionally, messages are provided for when relapse occurs and emergency messages are available to assist with cravings. This bank of motivational text messages was adapted in consultation with user groups to ensure they were appropriate for use in this setting. This involved user feedback and focus group testing with Aboriginal community members in collaboration with existing ACCHS partners and followed a previously used methodology to enhance applicability and acceptability to the target population.

Once the software specification was finalised the app was developed and deployed for access via Android and iOS operating systems. The app was made available for download via Google Play and Apple App Store.
