# Supplementary material for: A Smartphone App to Assist Smoking Cessation Among Aboriginal Australians: Findings From a Pilot Randomized Controlled Trial
Source: JMIR Mhealth Uhealth. 2019 Apr 2;7(4):e12745. doi: 10.2196/12745 (PMC6538311; doi:10.2196/12745)
Supplement: Multimedia Appendix 4 [file mhealth_v7i4e12745_app4.pdf]

Multimedia Appendix 3

Participant interview guide

**Area of Interest**	**Initial Questions**	**Possible Probing Questions**
**Smoking Habits**	**Tell me about your story of smoking?**	1. How did you get started smoking in the first place?<br>2. In what ways did family and friends influence your smoking<br>3. Can you tell me about ant quit attempts you have made?
**Smoking Cessation**	**What services/ treatments have you used in attempting to quit smoking?**	1. Have you used NRT to help your quit attempt? If so, what form of NRT have you used?<br>2. Have you ever been referred to a local smoking specific program, clinic or tobacco worker?<br>3. Have you been given smoking specific information to help you quit or been referred to Quitline?
**Mobile phone usage**	**Can you tell me about your day to day usage of mobile phones?**	1. What aspects of your phone do you use the most?<br>2. Is your mobile phone pre-paid or on a plan?<br>3. What are your favourite apps? (probe social media, games use)<br>4. Have you accessed health related apps previously?<br>5. In future would you use apps for health related situations?
**General Impact of the program**	**What was your overall impression of the Can't Even Quit app?**	1. How often would you say you used the app? (if not used ask about registration barriers)<br>2. What part of the app did you use more frequently?<br>3. Did you invite anyone else to use the app through the challenge function?<br>4. Do you have any feedback regarding the text messages?<br>5. Did you access the Emergency text messages?<br>6. Were there particular parts of the app that you did not like? (If so what were they?)<br>7. Are there any changes to the app that would make you use it more frequently?

**App Set-up**	**Did you experience any technical problems in using the app?**	1. Were there any issues you faced with downloading and using the app? (If so what were they?)<br>2. Did you have someone at the health centre to help you download it?<br>3. Do you have access to Wi-Fi at work or home?<br>4. Throughout the trial did the use of data restrict your use of the app?
**Trial experience**	**How did you find participating in the trial?**	1. Have you been in research trials previously?<br>2. How did you find out about the Can't Even Quit trial?<br>3. Did you understand what was involved in the trial when you signed up?<br>4. What was your overall impression of the follow up phone calls?<br>5. Do you have any suggestions regarding ways to improve the process?<br>6. Would you be part of another trial in the future? (general vs smoking trials)
**Final comments**		Is there anything else you would like to add that we have not discussed in this interview?
